# Janus regulation of ice growth by hyperbranched polyglycerols generating dynamic hydrogen bonding

**DOI:** 10.1038/s41467-022-34300-x

**Published:** 2022-11-01

**Authors:** Sang Yup Lee, Minseong Kim, Tae Kyung Won, Seung Hyuk Back, Youngjoo Hong, Byeong-Su Kim, Dong June Ahn

**Affiliations:** 1 KU-KIST Graduate School of Converging Science and Technology, Korea University, Seoul, Republic of Korea; 2 The w:i Interface Augmentation Center, Korea University, Seoul, Republic of Korea; 3 Department of Chemistry, Yonsei University, Seoul, Republic of Korea; 4 Department of Chemical and Biological Engineering, Korea University, Seoul, Republic of Korea

**Keywords:** Bioinspired materials, Surface assembly, Polymers

## Abstract

In this study, a new phenomenon describing the Janus effect on ice growth by hyperbranched polyglycerols, which can align the surrounding water molecules, has been identified. Even with an identical polyglycerol, we not only induced to inhibit ice growth and recrystallization, but also to promote the growth rate of ice that is more than twice that of pure water. By investigating the polymer architecture and population, we found that the stark difference in the generation of quasi-structured H_2_O molecules at the ice/water interface played a crucial role in the outcome of these opposite effects. Inhibition activity was induced when polymers at nearly fixed loci formed steady hydrogen bonding with the ice surface. However, the formation-and-dissociation dynamics of the interfacial hydrogen bonds, originating from and maintained by migrating polymers, resulted in an enhanced quasi-liquid layer that facilitated ice growth. Such ice growth activity is a unique property unseen in natural antifreeze proteins or their mimetic materials.

## Abstract (web summary)

Regulation of ice growth and ice growth inhibition are critical and different materials mimicking anti freeze proteins or ice binding surfaces have been developed. Here, the authors combine both concepts in a hyperbranched polyglycerols to change the ice growth activity.

## Introduction

Water freezing is a commonly observed natural phenomenon; however, ice growth and recrystallization can critically damage living organisms^1^. Nature has evolved to produce antifreeze proteins (AFPs)^2^ to survive this freezing threat. Their specific amino acid sequence has been widely accepted to play a critical role in binding to ice^3–9^, which can result in antifreeze activity when the Kelvin effect is dominant at the ice interface. To date, various studies mimicking the unique antifreeze activity of AFPs have been reported by using glycoproteins^10^, carbohydrates^11,12^, polymers^13–18^, supramolecules^19^, carbon materials^20–22^, and gold nanoparticles^23^. On the contrary, ice-binding surfaces can also lead to heterogeneous ice nucleation when the appropriate chemical and dimensional aspects are satisfied. Ice nucleation proteins and their mimics possess a large ice-binding surface, which facilitates the organization of surrounding water molecules in an ice-like lattice that could promote ice nucleation^24–30^. Cryosurgery utilizing such promoted ice growth within cells has been suggested^31,32^. The effects of surface size, shape, and material type on antifreeze or ice nucleation were investigated through computational analyses^33–36^. Both phenomena, which require ice-binding characteristics in common, demand distinct design protocols, and thus active materials have been developed by tailoring them for respective purposes. In this work, we report a single type of hyperbranched polymeric agent that exhibits both promotion and inhibition activities. The polymer’s formation–dissociation dynamics of hydrogen bonds (H-bonds) with the ice interface regulated ice growth in opposite tendencies depending on its population.

## Results

### Janus effect of polyglycerols on ice growth and recrystallization

We selected hyperbranched polyglycerol (*hb*PG) to realize the Janus effect on ice growth and recrystallization. Owing to its unique three-dimensional architecture, which comprises a polyether backbone with several functional hydroxyl groups, along with its excellent biocompatibility and immunogenicity^37^, *hb*PG has gained significant attention in biological and biomedical applications^38,39^. Moreover, their facile synthetic nature and access to various architectures (i.e., hyperbranched and linear with varying degrees of branching (DB)) have prompted us to investigate the interaction of PGs with the ice surface to form dynamic H-bonds. Accordingly, we synthesized *hb*PG and linear polyglycerol (*lin*PG) to study their effects on ice growth and recrystallization with respect to their architecture and concentration (details available in the Supplementary Figs. 1–5 and Supplementary Table 1).

**Fig. 1:**
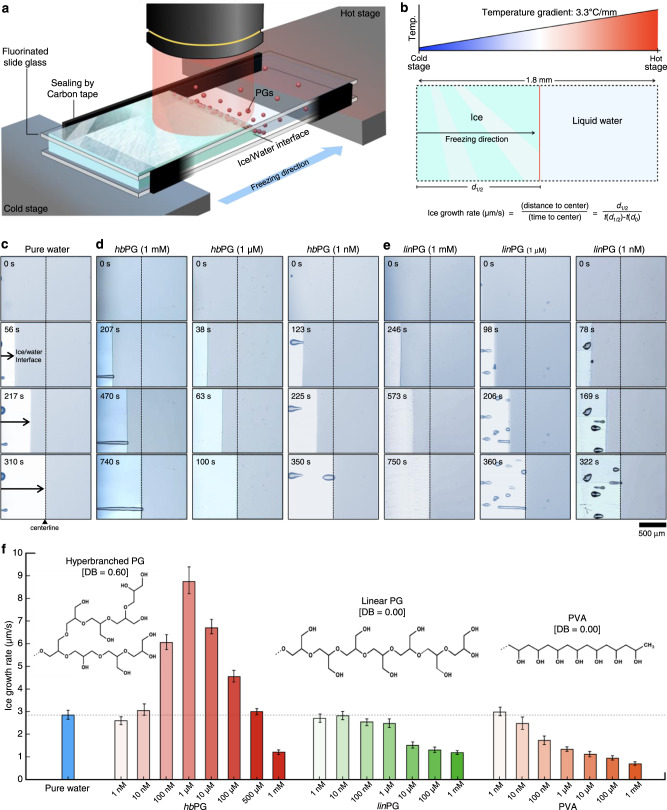
Evaluation of the effect of PGs on ice growth. **a** Schematic of the instrument for one-directional freezing experiments. **b** Temperature gradient according to the freezing coordinate and quantification of ice-growth rate. **c**, **d**, **e** Polarized optical and fluorescence microscopic images for the movement of the ice/water interface with respect to time in (**c**) pure water, (**d**) *hb*PG, and (**e**) *lin*PG. The status of the ice/water interface movement is shown after 0, 30, 60, and 90 s. The dotted lines and arrowhead marks indicate the centerline of the sample stage. **f** Ice-growth rate according to concentrations of PGs and PVA. Error bars indicate standard deviation.

We adopted a one-directional freezing method to determine the effects of the architecture and concentration of PGs on ice growth. As shown in Fig. 1a, we used a homemade instrument (Supplementary Fig. 6) to provide a temperature gradient along the freezing direction between a cold and a hot stage to observe the movement of the ice/water interface over time. The ice growth rate was obtained by calculating the velocity of the moving ice front to reach the centerline (Fig. 1b). We used *lin*PG (Mn = 8480 g mol^−1^, DB = 0.00) and *hb*PG (Mn = 8900 g mol^−1^, DB = 0.60) for the one-directional freezing experiments. The ice/water interface in pure water (Fig. 1c) reached the center position in ~310 s, which was faster than the *hb*PG solution at a concentration of 1 mM (Fig. 1d). Interestingly, the 1 μM solution required only ~100 s, which was ~3.1 times shorter than pure water. However, this promoting effect disappeared when the concentration was reduced to 1 nM. In contrast, in the case of *lin*PG, the time taken decreased monotonously with the concentration (Fig. 1e). The inhibitory activity on ice growth exhibited by *lin*PG resembled that of pure water upon dilution. Notably, the Cy5-conjugated PGs dissolved in water were clearly observable at the ice/water interfacial region: the PG molecules were pushed along the ice front and showed interface movement (Supplementary Fig. 7). The ice-growth rates for the two types of PGs and poly(vinyl alcohol) (PVA) with their concentrations are presented in detail in Fig. 1f, along with that of pure water, whose rate was estimated to be 2.85 ± 0.21 μm/s. The *hb*PG solution exhibited dramatic variations in ice-growth rate: 2.61 ± 0.32 μm/s at 1 nM, 8.74 ± 0.64 μm/s at 1 μM (3.1 times faster than pure water), and 1.22 ± 0.06 μm/s at 1 mM (more than two times slower than pure water). Interestingly, the rate at 0.5 mM resembled those of more dilute solutions under 10 nM. In contrast, *lin*PG exhibited a rather monotonic decrease in the ice-growth rate over the entire range of concentrations. PVA, a well-known polymer that exhibits some ice recrystallization inhibition (IRI) activity, exhibited a decreasing trend resembling that of *lin*PG (Supplementary Fig. 8). It is noteworthy that *hb*PG can play the opposing roles of promoter and inhibitor, which has not been observed in other polymeric materials to date.

**Fig. 2:**
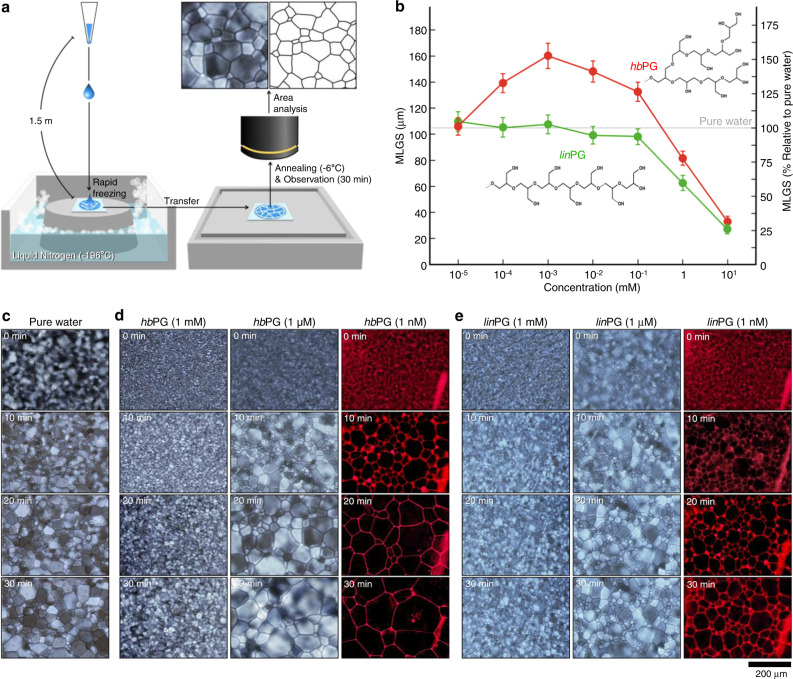
Evaluation of the effect of PGs on ice recrystallization. **a** Schematic of the experimental procedure for measuring ice recrystallization using the splat method. **b** Experimental results of ice recrystallization inhibition (RI) of two types of PGs. RI from *hb*PG (red symbols) and *lin*PG (green symbols) according to their molecular concentrations. **c**, **d**, **e** Time-traced cross-polarized optical microscopic images of **c** pure water, **d** *hb*PG, and **e** *lin*PG during ice recrystallization. Corresponding fluorescence images of the ice domain with Cy5-conjugated *hb*PG (**d**, right panels) and *lin*PG (**e**, right panels). Cy5-conjugated PGs, which were located at the interface between the ice domains, moved along the interfacial locus over time. The scale bars represent 200 µm. Error bars indicate standard deviation.

Further, we investigated the effect of PGs on ice recrystallization (Fig. 2a), which commonly occurs during the thawing of rapidly frozen cells. To explore the fundamental control of natural freezing, we performed the IRI assays in pure water which does not form a eutectic state. Figure 2b shows the IRI activity of *hb*PG and *lin*PG with respect to the concentration. The mean largest grain sizes (MLGS) of 1 mM *hb*PG was 81.5 ± 6.8  μm, indicating its ability to inhibit ice recrystallization. When its concentration decreased to 1 μM, the MLGS increased to 160.2 ± 12.1 μm, indicating that ice recrystallization was promoted compared to pure water, which is correlates with the enhanced ice-growth rate at 1 μM as described in Fig. 1. Upon further dilution, it converged to that of pure water. In contrast, *lin*PG showed an inhibitory activity of recrystallization with an MLGS of 61.7 ± 7.3 μm at 1 mM, but gradually converged upon dilution to that observed with pure water. In the microscopic image (Fig. 2c), pure water initially showed tiny ice grains with a size of several micrometers, and large ice grains of approximately 100 μm were observed after 30 min of ice recrystallization. The 1 mM *hb*PG (8.9 mg ml^−1^) solution contained tiny ice grains and no noticeable change was observed over 30 min, while the 1 μM solution facilitated recrystallization significantly and the size of ice grains increased to more than twice that of pure water (Fig. 2d). It is noteworthy that *hb*PG can play the promoter of recrystallization in pure water, which is an unexpected result when compared to other macromolecules that act only as inhibitors. At high concentrations (i.e., above 0.5 mM), *hb*PG inhibits the ice recrystallization by preventing the translocation of H_2_O molecules at grain boundary. Unlike *lin*PG, which showed a consistent inhibitory effect (Fig. 2e), *hb*PG exhibited the Janus effect again on ice recrystallization, which could be further modulated with different DB values (Supplementary Fig. 9). *hb*PG, used in the present study, showed the Janus effect at concentrations less than 0.01X PBS buffer solution.

### Molecular dynamics analyses of ice growth and water immobility by PGs

**Fig. 3:**
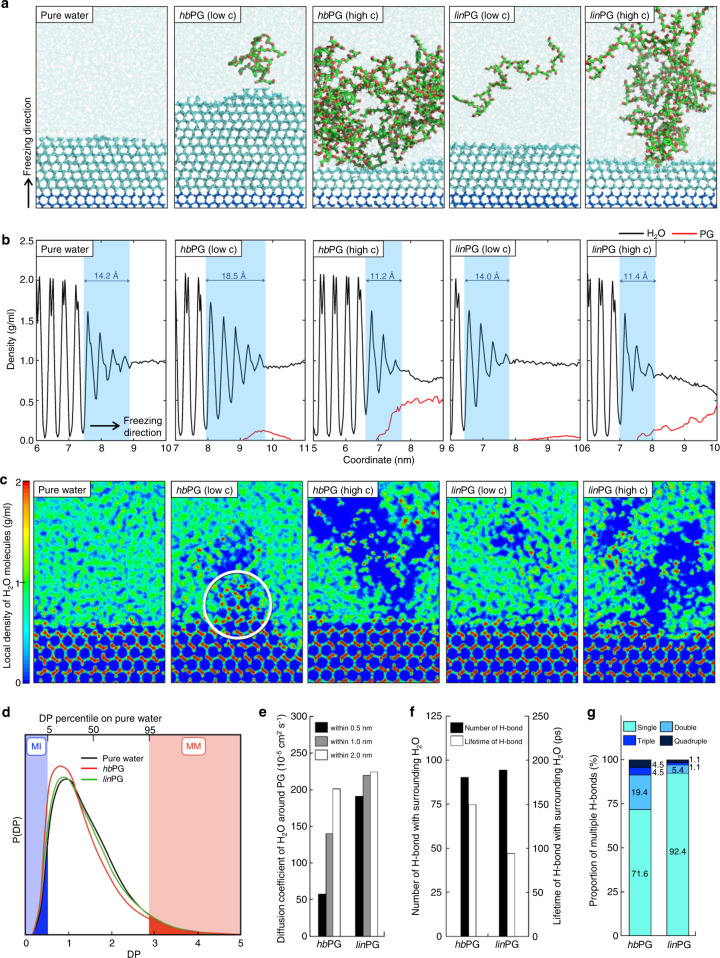
Molecular dynamic behavior of PGs with respect to the DB and molecular concentrations. Liquid water, growing ice, and PGs were represented by transparent sky blue, plain sky blue, and green colors, respectively. **a** Final configuration of ice-growth simulation of pure water (first column), *hb*PG at low (second column) and high (third column) concentrations, and *lin*PG at low (fourth column), and high (fifth column) concentrations. **b** Density profiles of water molecules (black line) and PGs (red line) along the freezing direction. The selected region (blue area) represents the region of the quasi-liquid layer. **c** Two-dimensional density mapping of the water molecules. The red and green colors represent the highest local density (2 g/cm^3^) and 1 g/cm^3^ of local density, respectively. The blue region in the liquid water is a low-density region owing to the presence of PG molecules. In the ice layer, the vacuum (low-density region) arises from the lattice of H_2_O molecules. The quasi-ordered structure of water molecules is indicated by a white circle in *hb*PG (low c). **d** DP distribution for pure water (black line), *hb*PG (red line), and *lin*PG (green line) In the DP distribution for pure water, the lower 5% is the most immobile (MI) region (blue area), and the top 5% is the most mobile (MM) region (red area). **e** Diffusion coefficient of the surrounding water molecules according to the distance from the PG molecule. **f** H-bonding analysis in terms of the average number and lifetime of the H-bonds. **g** Proportion of multiple H-bonds between PG and its surrounding water molecules.

To determine the mechanism of the Janus effect of *hb*PG on ice growth, we constructed one-directional ice growing simulations using atomistic molecular dynamics (MD) modeling (Supplementary Movies 1, 2). As shown in Fig. 3a, five systems, including pure water and two types of PGs with two molecular concentrations (i.e., single and ten PG molecules), were simulated. Pure water crystallized from the basal plane of the ice seed at 267 K, forming seven ice layers in 300 ns. A total of 12 layers of ice were formed in the systems with a low concentration of *hb*PG under the same conditions, resulting in an ice-growth rate 1.7 times faster than that of pure water. A high-concentration system with ten *hb*PG molecules was evaluated to elucidate the differences based on the concentration. In the presence of high-concentration *hb*PG, only three ice layers were grown from the ice seed, and no additional ice growth was observed. A low-concentration *lin*PG formed five ice layers, indicating that the ice-growth rate was 0.7 times slower than that of pure water. In the high-concentration system, *lin*PG formed a single ice layer, similar to the corresponding *hb*PG case. It is noted that the binding Gibbs free energy of *hb*PG and *lin*PG was determined to be −14.9 kcal/mol and −13.1 kcal/mol, respectively (Supplementary Fig. 10). Notably, the low-concentration *hb*PG surpassed pure water in ice growth, which matches the results of the one-directional ice growth and recrystallization experiments. This ice-growth promotion was found to be common for the prismatic plane (Supplementary Fig. 11). In addition, such promotion was enhanced as the DB of *hb*PG increased (Supplementary Fig. 12).

We observed the structural properties of the ice/water interface from the perspective of the quasi-liquid layer (QLL) to investigate the cause of the Janus ice growth. As shown in Fig. 3b, the density profiles of the five systems were calculated along the freezing direction. During ice growth, the QLL thickness of pure water was of 14.2 ± 0.3 Å, which was maintained throughout the simulation. When the low-concentration *hb*PG was located on the ice surface, the QLL thickness increased to 18.5 ± 0.4 Å. In contrast, the high-concentration *hb*PG reduced the thickness to 11.2 ± 0.1 Å. For *lin*PG, thicknesses of 14.0 ± 0.3 Å and 11.4 ± 0.1 Å were observed at the low concentration, similar to pure water, and high concentration, respectively. Hence, we can state that the extent of ice growth is highly correlated with the thickness of the QLLs. In order to figure out the molecular arrangement of H_2_O molecules in the QLL, we applied a two-dimensional density colormap (Fig. 3c). When *hb*PG is located at the ice/water interface, it could form high-density regions having ice-like structures just above the ice surface. The pronounced structuring of water molecules by *hb*PG at the low concentration contributed to the large QLL thickness of 18.5 Å.

The formation of quasi-ordered water molecules can be attributed to their restricted movement, just like ice, which would then relate quantitatively to their mobility. We analyzed the mobility in terms of the dynamical propensity using iso-configurational analysis (ISOCA)^40–42^. In the presence of *hb*PG, the amount of water molecules in the most immobile (MI) region increased by 28% compared to pure water and *lin*PG (Fig. 3d). Thus, *hb*PG drastically slowed the surrounding water molecules. The MI region has been reported to play an essential role in ice-like ordered structure^42^. Next, we analyzed the diffusion coefficient of water molecules according to the distance from the PG (Fig. 3e). Around *hb*PG, water exhibited various diffusion coefficients of 53 × 10^−3^, 146 × 10^−3^, and 205 × 10^–3^ nm^2^/s at distances of 0.5, 1.0, and 2.0 nm, respectively. On the other hand, *lin*PG showed the coefficients from 181 × 10^−3^ to 224 × 10^–3^ nm^2^/s, slightly increased with distance. The reduced mobility of the surrounding water molecules is considered to be related to the H-bonding capability of *hb*PG. In addition, such reduced mobility was proved experimentally by NMR spin–spin relaxation (T_2_) measurements, which indicated that the water molecules around the *hb*PG were more confined than those of *lin*PG (Supplementary Fig. 13 and Supplementary Table 2). As shown in Fig. 3f, the H-bond had a longer lifetime of 151 ps with *hb*PG compared to 97 ps with *lin*PG, despite the similar number of H-bonds in both cases. Figure 3g shows that the proportion of multiple H-bonds, including double, triple, and quadruple, was 28.4% for *hb*PG and 7.6% for *lin*PG, indicating the former forms 3.7 times more multiple H-bonds than the latter. This multiplicity can be intensified effectively as the ether groups in the PGs are localized more in the internal cavities when hyperbranched (Supplementary Fig. 14). The distribution of ether groups is related to the stabilized structure of PGs in an aqueous solution. The hydrodynamic size and volume of PGs, measured experimentally by dynamic light scattering (DLS) and ^1^H diffusion-ordered NMR spectroscopy (DOSY), were maintained in the range of concentrations in the present study (Supplementary Figs. 15,  16). Likewise, in Supplementary Fig. 17, *lin*PGs were calculated to possess large end-to-end distance and radius of gyration in aqueous media. On the other hand, *hb*PG maintained its hyperbranched globular structure, allowing the accommodation of neighboring water molecules for multiple H-bonds. This promotes the generation of quasi-ordered structures, as indicated by the larger QLL acting as the MI region for ice growth.

### Time-traced path of water molecules and action of *hb*PG on ice growth

**Fig. 4:**
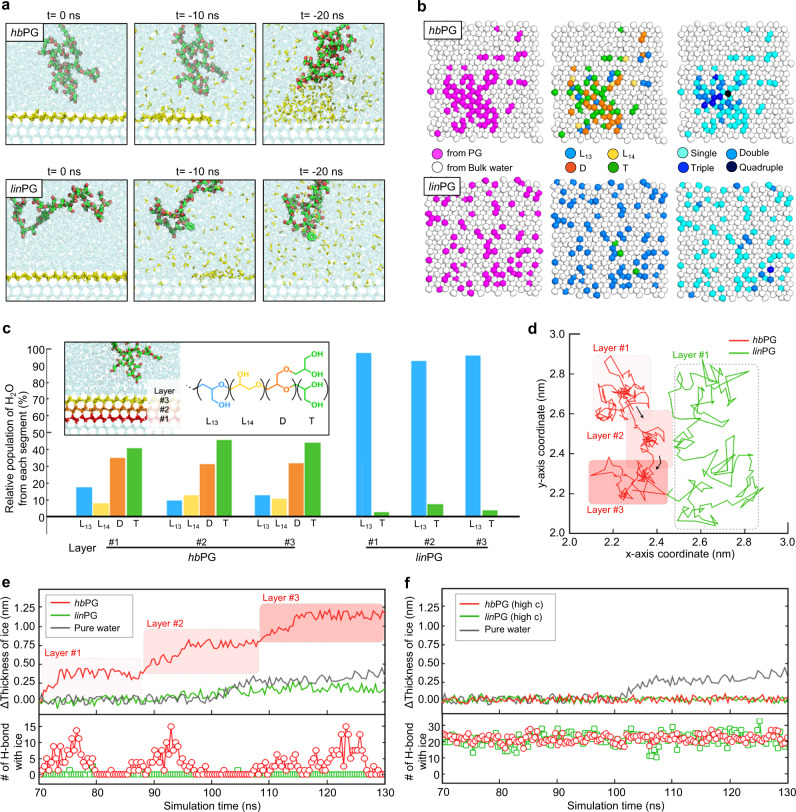
Back-tracking analysis to identify the path of frozen water. **a** Snapshots of the final configurations of the ice-growth simulations in *hb*PG (**a**, upper panels) and *lin*PG (**a**, lower panels). Preselected water molecules are represented in yellow. **b** The lateral colormap is classified according to molecular type (**b**, left panels), polymer unit group (**b**, center panels), and the type of bonding-bonding (**b**, right panels). In the classification by molecular types, H_2_O molecules from PG and bulk water are colored magenta gray, respectively. In the classification by the unit group of polymers, L_13_, L_14_, D, and T are represented by blue, yellow, orange, and green van der Waal’s spheres, respectively. When classified according to the multiplicity of H-bonds formed with PG, the color of the spheres becomes darker blue at higher degree of multiple H-bonding. **c** Repeated back-tracking analysis for three consecutive layers. The left inset image represents the selected three ice layers, and the right inset shows segment information in the molecular structure of PG. **d** Time-traced lateral movement of dilute *hb*PG (red line) and *lin*PG (green line) between 70 ns and 130 ns. **e** Changes in ice thickness and number of H-bonds formed with ice during the same period of **d**. (For *lin*PG, ice grew 1.1 nm in thickness until 250 ns.) **f** Same as **e**, but at higher concentrations of *hb*PG and *lin*PG.

Furthermore, we performed a back-tracking analysis to trace the paths of water molecules frozen in ice (Supplementary Movies 3, 4). As shown in Fig. 4a, we selected a layer of frozen H_2_O molecules and reverse-regenerated the fully-grown structure for 20 ns. In the presence of *hb*PG, a large portion of the H_2_O molecules were located between *hb*PG and the ice surface in the 20 ns past. In contrast, an identical analysis of *lin*PG revealed that most of the water molecules were scattered throughout the bulk water. Based on the back-tracking results, we probed the molecular paths sequentially from liquid water to ice in terms of the following three characteristics (Fig. 4b): H_2_O molecules experiencing (1) travel via PG, (2) travel via specific segments of PG, and (3) multiple H-bonding. In the left panels of Fig. 4b, H_2_O molecules are classified according to their travel path through the PG. In the case of *hb*PG, 23% of H_2_O molecules traveled via *hb*PG and were mostly localized around *hb*PG. Likewise, 25% traveled via *lin*PG, but were randomly distributed. The center panels show that these H_2_O molecules traveled through four segments of PG: primary alcohol L_13_, secondary alcohol L_14_, terminal group T, and dendrimer D units. In *hb*PG, the H_2_O molecules that turned ice predominantly underwent H-bonding with the T and D units. In *lin*PG, most of the H_2_O molecules traveled through L_13_. As for the multiplicity of H-bonding shown in the right panels, compared to *lin*PG, *hb*PG formed more multiple H-bonds with H_2_O molecules (Supplementary Fig. 18), which consequently localized on the ice surface.

We repeated the same analysis for the underlying consecutive ice layers (Fig. 4c). For each of the three layers denoted as layers #1, #2, and #3 from the bottom, the relative proportions of the paths of the water molecules before turning ice were analyzed. Approximately 76% of H_2_O molecules via *hb*PG traveled through T and D, that is, 40–46% via T and 31–35% via D. However, in the case of *lin*PG, more than 90% of the H_2_O molecules passed through L_13_. In Fig. 4d, we tracked the lateral movement of *hb*PG during the formation of three ice layers between 70 and 130 ns and compared it with that of *lin*PG during the same time period. *hb*PG underwent sporadic migration in the lateral direction, leading to reduced mobility at local positions while *lin*PG continued to move actively. The increase in ice thickness was then calculated with respect to the type of polymer (Fig. 4e, top). In *hb*PG, the ice grew stepwise, that is, the height increased by 0.37 nm at 87, 108, and 130 ns. This height change of 0.37 nm is equal to the thickness of one layer of the basal plane (measured using electron diffraction and atomic force microscopy^43,44^), indicating the three ice layers were promoted to grow in 130 ns in our simulation. In contrast, no stepwise growth occurred in *lin*PG: it grew to 0.19 nm in 130 ns and then to 1.10 nm in 250 ns. It is interesting to note that the stepwise growth of ice in the presence of *hb*PG is evidently related to the formation of H-bonds with the ice surface (Fig. 4e, bottom). The number of H-bonds of *hb*PG with ice increased to 15 and then decreased to 0, and this pattern was repeated over time. The increase in H-bonds conformed to the moment when atop ice layer began to grow (Supplementary Fig. 19). Then, with the breaking of the H-bonds (Supplementary Fig. 20), the polymer began to migrate laterally until it settled elsewhere. In contrast, *lin*PG, moving actively, formed only a few H-bonds and did not exhibit any peculiar pattern. Concentrated *hb*PG and *lin*PG (Fig. 4f) maintained approximately 22 H-bonds per polymer near the ice surface during the inhibiting ice growth (Supplementary Fig. 21), and the polymers were found to adhere nearly at fixed loci (Supplementary Fig. 22). In addition, concentrated PGs significantly lower the mobility of surrounding water molecules and restrain the migration of them, thereby exhibiting inhibitory activity (Supplementary Fig. 23). The concentrated PGs maintained a large number of hydrogen bonds with ice surface; however, they did not bind to any specific plane of ice crystals (Supplementary Fig. 24) like certain molecular weight ranges of polymers of PVA.

In summary, *hb*PGs exhibited the Janus behavior of promoting or inhibiting ice growth and recrystallization, regulated by their concentration, while *lin*PG displayed only inhibition. Our combined experimental and theoretical investigations revealed that the PGs, whose molecular structures differed according to their DB, affected the multiplicity in H-bonding with neighboring water molecules. Among them, *hb*PG most efficiently formed multiple H-bonds, making the H_2_O molecules more immobile and further inducing quasi-ordered H_2_O and thicker QLLs. It is noteworthy that during migration, *hb*PG molecules repeated the pattern of formation and dissociation of H-bonds with ice, promoting stepwise ice growth with time. However, this dynamic formation disappeared at higher concentrations of *hb*PG, and inhibitory activity was manifested owing to the maintenance of numerous H-bonds with ice. Going beyond the existing materials focused on inhibiting ice growth, we presented a design strategy that enables a wider control of water freezing activity ranging from inhibition to promotion, even with the use of a single type of polymeric material. This discovery is expected to deepen the fundamental understanding of ice growth and affect current technological applications that require ice control.

## Methods

### Reagents and analyses

^1^H and ^13^C NMR spectra were recorded at 298 K with an Agilent 400 MHz spectrometer equipped at ambient using CDCl_3_ and D_2_O solvents. All spectra were recorded in ppm units with tetramethyl silane (TMS) as an internal standard in the deuterated solvents. Gel permeation chromatography (GPC) measurements (Agilent 1200 series) were performed using DMF as an eluent at 40 °C with a flow rate of 1.0 mL min^−1^ using a refractive index detector. GPC instrument equipped with PLgel 5 µm guard and two PLgel 5 µm mixed-D columns (Agilent). Standard poly(ethylene oxide) (PEO) samples were used for calibration to determine the number- and weight-averaged molecular weight (*M*_n_ and *M*_w_). All solvents and reagents were purchased from commercial sources (Sigma-Aldrich, TCI, and Alfa Aesar). Ethoxyethyl glycidyl ether (EEGE) was prepared. *N*-methyl-2-pyrrolidone (NMP), EEGE, and glycidol were purified by vacuum distillation over calcium hydride (CaH_2_) prior to use.

### General synthesis procedure of hyperbranched P(G-*co*-EEGE)s

All glassware was washed and flame-dried before polymerization. A 0.11 mL solution of *t*-BuP_4_ (0.8 M, 0.09 mmol) in n-hexane was added to the solution of benzyl alcohol (10.67 μL, 0.1 mmol) in NMP under an N_2_ atmosphere. Then, 10 mmol of glycidol, EEGE, or glycidol/EEGE mixture solution (monomer: NMP ratio = 1:1 (v/v), the total volume of NMP was set to 2.4 mL) was added dropwise to the initiator solution for 24 h and then stirred for 6 h. After polymerization was completed, the reaction mixture was quenched with excess methanol. The polymer in methanol solution was passed through an Amberlite IR-120(H) ion exchange resin twice to remove the phosphazene base. The polymer solution was then vacuum distilled to remove the remaining NMP to obtain pale yellow transparent polymers of P(G-*co*-EEGE) s. Successful polymerization of P(G-*co*-EEGE) was confirmed by ^1^H NMR and GPC. ^1^H NMR (400 MHz, CDCl_3_) δ 4.70 (s, 60H), 4.53 (s, 2H), 3.97–3.26 (m, 628H), 1.24–1.29 (m, *J* = 28.8 Hz, 364H). (*M*_n,NMR_: 11840; *M*_n,GPC_: 11320; *M*_w_/*M*_n_: 1.23) N_3_ initiated PGs were polymerized from 6-azido-1-hexanol.

### General hydrolysis procedure of P(G-*co*-EEGE)

A 10 wt.% *hb*P(G-*co*-EEGE) solution in a 1:1(v/v) mixture of 1 M HCl and MeOH was stirred at 40 °C for 24 h. After removing the solvent, the polymer was dissolved in MeOH and precipitated twice in cold diethyl ether. The polymer solution was concentrated in vacuo and further dried at 60 °C for 24 h to obtain transparent pale yellow *hb*PGs (yield: 28–40%). The successful hydrolysis of P(G-*co*-EEGE) was confirmed by various characterization techniques, including ^1^H and inverse-gated ^13^C NMR, GPC. ^1^H NMR (400 MHz, D_2_O) δ 7.39 (s, 5H), 4.55 (s, 1H), 3.98–3.42 (m, 408H), ^13^C NMR (101 MHz, D_2_O) δ 79.61, 78.23, 72.16, 70.63, 69.01, 62.66, 60.83. (*M*_n,NMR_: 6120; *M*_n,GPC_: 3210; *M*_w_/*M*_n_: 1.25)

The *hb*PGs were characterized by inverse-gated ^13^C NMR spectroscopy (Supplementary Fig. 2), and the degree of branching (DB) was calculated by integrating the values from each carbon unit. The formula for calculating DB is:

1 $${{{{{\bf{DB}}}}}}\,=\,\frac{{{{{{\bf{2}}}}}}{{{{{\boldsymbol{D}}}}}}}{{{{{{\bf{2}}}}}}{{{{{\boldsymbol{D}}}}}}\,+\,{{{{{{\boldsymbol{L}}}}}}}_{{{{{{\bf{13}}}}}}}\,+\,{{{{{{\boldsymbol{L}}}}}}}_{{{{{{\bf{14}}}}}}}}$$

D is dendritic, L is linear, and L_13_ and L_14_ are the methine carbons in the PG backbone, which are exposed to primary and secondary alcohols, respectively. Varying the glycidol ratio, it was confirmed that the ratio of D units and DB increased from 0 to 32% and 0 to 0.60, respectively.

As the DB increased, the ratio of the L_14_ unit increased from 0 to 24%, the L_13_ unit decreased from 96 to 12%, while the T unit increased from 4 to 32% (Supplementary Fig. 4). DB is highly correlated with the three-dimensional size and structure of PG. As DB increased, the PGs became smaller and spherical. Hence, although the molecular weights of PGs are similar in NMR (Table S1), *hb*PGs with high DB exhibit a lower molecular weight distribution in GPC than *lin*PG because its hydrodynamic radius is smaller than that of *lin*PG (Supplementary Table 1 and Fig. 2).

### One-directional ice-growth experiment

We designed a temperature-controlled stage based on two Peltier elements that can both heating and cooling. For controlling temperature, we chose two Peltier modules (FPK2-19808NC, Z-max Co.). Each Peltier module was controlled using a PID Controller (OPS-305, ODA Co.) that was operated with LabVIEW software. The temperature measurements of the two stages was acquired independently, and the temperature precision/stability was <0.1 °C. The sample holder, a Hele-Shaw cell, consisted of a sandwich of two glass slides separated by an 80 µm gap. The sides of the sample holder were sealed with carbon tape, and the exterior surface was fluorinated to prevent heat loss and allow heat to flow from the hot to the cold stage. Polarized optical and fluorescence microscopes were used to observe the movement of the ice/water interface over time. The experiments proceeded with the sample holder located at the fixed position in the middle of the temperature-controlled stage. Upon nucleation on the cold side, ice-growth proceeded so that the ice/water interface formed a straight line and moved towards the hot stage throughout the growth experiments in this study. Maintaining flat ice interface in our set-up was enabled in the operation window having cold-block temperature between −5 °C and −15 °C and hot-block temperature between 5 °C and 15 °C. In this study, the temperature gradient was 3.33°C/mm by setting cold-block at −5 °C and hot-block at 5°C. The ice-growth rates obtained by using this method, operated at the above temperature gradient, were in good agreement with those by the conventional moving-stage method. The present set-up afforded a simple way to observe the Janus regulation phenomenon of ice growth. Each experiment was repeated three times or more, and the ice growth rate was calculated using the following equation (Fig. 1b):

2 $${{{{{{\boldsymbol{V}}}}}}}_{{{{{{\bf{ice}}}}}}\,{{{{{\bf{growth}}}}}}\,{{{{{\bf{rate}}}}}}}({{{{{\boldsymbol{\mu }}}}}}{{{{{\boldsymbol{m}}}}}}/{{{{{\boldsymbol{s}}}}}})=\frac{{{{{{\bf{distance}}}}}}\,{{{{{\bf{to}}}}}}\,{{{{{\bf{center}}}}}}}{{{{{{\bf{time}}}}}}\,{{{{{\bf{to}}}}}}\,{{{{{\bf{center}}}}}}}=\frac{{{{{{{\boldsymbol{d}}}}}}}_{{{{{{\boldsymbol{1}}}}}}/{{{{{\boldsymbol{2}}}}}}}}{{{{{{\boldsymbol{t}}}}}}({{{{{{\boldsymbol{d}}}}}}}_{{{{{{\boldsymbol{1}}}}}}/{{{{{\boldsymbol{2}}}}}}}-{{{{{{\boldsymbol{d}}}}}}}_{{{{{{\boldsymbol{0}}}}}}})}$$

### Ice recrystallization experiment

For RI activity, a splat-freezing method was used. The 10 µL sample droplets were rapidly frozen by dropping on a precooled stage, which was cooled using liquid nitrogen (i.e., −196 °C). The frozen samples were subsequently annealed at −6 °C on a temperature-controlled microscope stage and the transition through recrystallization observed for 30 min. After 30 min, the average size of the 10 largest ice domains was estimated, and the MLGS was evaluated by comparing it with the size obtained with pure water. The MLGS presented in the main text were calculated using four observations.

### NMR spin–spin relaxation (T2) measurements for water in PGs samples

The proton spin–spin relaxation time (T2) measurements of water in PGs samples were carried out on a 400 MHz NMR spectrometer (400JJYH, ZEOL) using the Carr-Purcell-Meiboom-Gill (CPMG) pulse sequence. A tau interval of 1.2 ms between the 90° and 180° pulse was used, and the spin-lattice relaxation time between successive scans was set sufficiently (at least 10 times) to fully recover the magnetization between acquisitions^45^. The PG solution mixed with D_2_O (volume ratio of 1:99) was filled into the NMR tube. All the T_2_ relaxation behaviors for the water proton were fitted with a biexponential decay curve.

3 $${{{{{{\boldsymbol{E}}}}}}}_{{{{{{\boldsymbol{t}}}}}}}\,=\,{{{{{{\boldsymbol{f}}}}}}}_{2,{{{{{\boldsymbol{a}}}}}}}\exp (-\frac{{{{{{\boldsymbol{t}}}}}}}{{{{{{{\boldsymbol{T}}}}}}}_{2,{{{{{\boldsymbol{a}}}}}}}})\,+\,{{{{{{\boldsymbol{f}}}}}}}_{2,{{{{{\boldsymbol{b}}}}}}}\exp (-\frac{{{{{{\boldsymbol{t}}}}}}}{{{{{{{\boldsymbol{T}}}}}}}_{2,{{{{{\boldsymbol{b}}}}}}}})\,+\,{{{{{{\boldsymbol{E}}}}}}}_{0}$$

The dynamic of water can be represented by the correlation time for the motion of water (τ_c_) by using the Bloembergen Purcell and Pound equation as

4 $$\frac{1}{{{{{{{\boldsymbol{T}}}}}}}_{2}}\,=\,\frac{{{{{{\boldsymbol{C}}}}}}}{2}(3{{{{{{\boldsymbol{\tau }}}}}}}_{{{{{{\boldsymbol{c}}}}}}}\,+\,\frac{5{{{{{{\boldsymbol{\tau }}}}}}}_{{{{{{\boldsymbol{c}}}}}}}}{1\,+\,{{{{{{\boldsymbol{\omega }}}}}}}_{0}^{2}{{{{{{\boldsymbol{\tau }}}}}}}_{{{{{{\boldsymbol{c}}}}}}}^{2}}\,+\,\frac{5{{{{{{\boldsymbol{\tau }}}}}}}_{{{{{{\boldsymbol{c}}}}}}}}{1\,+\,4{{{{{{\boldsymbol{\omega }}}}}}}_{0}^{2}{{{{{{\boldsymbol{\tau }}}}}}}_{{{{{{\boldsymbol{c}}}}}}}^{2}})$$

where *C* is a constant for water of 5.33 × 10^9^ s^−2^ and *ω*_0_ is the Larmor frequency^46^.

### ^1^H DOSY NMR experiments

Two samples (*hb*PG and *lin*PG) were separately prepared at a concentration of 1.0 mM in D_2_O. For diffusion measurements, pulsed-field-gradient DOSY NMR experiments were performed with a maximum gradient of 52.9 mT m^−1^. The pulse sequences contained a 2.0 ms delay to stabilize the gradients. The magnetic field gradient amplitudes were gradually increased from 2.10 × 10^−2^ up to 5.2 × 10^−1^ T m^−1^ for the maximum gradient strength in a linear ramp. For each gradient amplitude, 16 transients of 16384 complex data points were obtained for a total experimental time of 16 min. The diffusion gradient length (δ) of 2 ms was selected for the diffusion time, with a diffusion delay of 20 ms. The NMR data were processed, and the diffusion coefficients (D) were determined using the DOSY Toolbox software package. Errors of approximately 1.8% were obtained for the diffusion experiments. The main source of error in the diffusion experiments was the reproducibility of data acquisition. The signal decay caused by gradients was obtained by DOSY fitting of the Stejskal–Tanner equation:

5 $${{{{{\boldsymbol{S}}}}}}\,=\,{{{{{{\boldsymbol{S}}}}}}}_{0}\exp ({{{{{\boldsymbol{D}}}}}}\,-\,{{{{{{\boldsymbol{\gamma }}}}}}}^{2}{{{{{{\boldsymbol{G}}}}}}}^{2}{{{{{\boldsymbol{\delta }}}}}}(\varDelta \,-\,\frac{{{{{{\boldsymbol{\delta }}}}}}}{3}))$$

where *S* is the signal amplitude as a function of gradient strength *g*, *S*_*0*_ is the signal amplitude at *g* = 0, *D* is the diffusion coefficient, *γ* is the proton gyromagnetic ratio, *δ* is the gradient pulse duration, and *Δ* is the diffusion time.

### System parameters of molecular dynamics simulation

All simulations were performed using the GROMACS^47^ package (version 5.1.4) and the CHARMM general force field (CGenFF)^48^ for all-atomic (AA) modeling. The TIP4P-ice water model, which can simulate the water-ice phase transition well, was used^49^. To control the temperature, a V-rescale^50^ was used as the thermostat for equilibrium. The pressure was maintained at 1 bar using a Berendsen^51^ and Parrinello-Rahman^52^ barostat for the equilibrium and production runs, respectively. Neighbor lists were built using the Verlet cutoff scheme with a cutoff radius of 1.2 nm. The linear constraint solver (LINCS)^53,54^ algorithm was used to constrain the bond lengths. All simulations were performed using a leapfrog integrator with time steps of 2 fs during 150,000,000 steps (total 300 ns) for ice-growth simulation. Electrostatic interactions were calculated using particle mesh Ewald (PME)^55^ with a cutoff of 1.2 nm in AA-MD.

### AA modeling of PG molecules

PG molecules were parameterized based on the CHARMM36 force field (version mar-2019) parameters. Molecular structures were designed based on experimental NMR results (Supplementary Fig. 4) to match the ratio of the PG segments according to the DP. To consider the randomness of the branching structure of *hb*PG, a molecular model with a different distribution of segments was used in the repeat calculations (a total of five models were used, one for each repeat system). In the high-concentration simulation, a system was constructed by inserting two molecules of five models for a total of 10 PG molecules.

### System design for ice-growth simulation

Ice-growth simulations were conducted by placing an ice seed at the bottom of the system and allowing ice to grow from the bottom up at a temperature of 268 K. The PG molecule was initially placed 2 nm above the ice seed to prevent it from interacting with the seed. Three types of ice surfaces were used for the ice contacts with the PG: basal, primary prism, and secondary prism. The ice-growth simulation was repeated four times considering the type, structure, and initial configuration of the PG molecules.

### Back-tracking analysis

To analyze the factors affecting the water molecules that formed ice, ice-growth simulation was reverse-generated, and the hydrogen bonding was analyzed. A time period of 20 ns was selected for the complete growth of one ice layer. Water molecules constituting the flat ice layer were preselected and reverse-regenerated up to −20 ns to track the movement of water molecules. The trajectory of the simulation conducted for 20 ns was subdivided into 2000 frames at 100 ps intervals. Hydrogen bonding was analyzed in each frame and classified by the presence or absence of hydrogen bonding with PG and its types (i.e., H-bonding multiplicity) were recorded.

## Supplementary information

Supplementary Information

Description of Additional Supplementary Files

Supplementary Movie 1

Supplementary Movie 2

Supplementary Movie 3

Supplementary Movie 4

## Data Availability

All relevant data are available from the authors upon request. Source data are provided in this paper. Source data are provided with this paper.

## Source data

Source Data
