# Supplementary material for: Janus regulation of ice growth by hyperbranched polyglycerols generating dynamic hydrogen bonding
Source: Nat Commun. 2022 Nov 1;13:6532. doi: 10.1038/s41467-022-34300-x (PMC9626502; doi:10.1038/s41467-022-34300-x)
Supplement: Supplementary file 1 — Supplementary Information [file 41467_2022_34300_MOESM1_ESM.pdf]

Supplementary Information for

Janus Regulation of Ice Growth by Hyperbranched Polyglycerols Generating Dynamic Hydrogen Bonding

Sang Yup Lee[1,2,5], Minseong Kim[3,5], Tae Kyung Won[2,4,5], Seung Hyuk Back[2,4], Youngjoo Hong[3], Byeong-Su Kim[3*] & Dong June Ahn[1,2,4*]

[1]KU-KIST Graduate School of Converging Science and Technology, Korea University; Seoul 02841, Republic of Korea
[2]The w:i Interface Augmentation Center, Korea University; Seoul 02841, Republic of Korea
[3]Department of Chemistry, Yonsei University; Seoul 03722, Republic of Korea
[4]Department of Chemical and Biological Engineering, Korea University; Seoul 02841, Republic of Korea
[5]These authors contributed equally to this work

**Corresponding author. Email: ahn@korea.ac.kr (D.J.A.); bskim19@yonsei.ac.kr (B.-S.K.)*

This PDF file includes:

Supplementary Fig. 1 Synthesis of polyglycerol with controlling DB
Supplementary Fig. 2 GPC traces of *lin*PG and *hb*PGs
Supplementary Fig. 3 Inverse-gated ^{13}C NMR of PGs
Supplementary Fig. 4 Ratio of each methine groups on PGs
Supplementary Fig. 5 Characterization of Cy5-conjugated PG
Supplementary Fig. 6 Homemade instrument and environmental conditions for unidirectional ice growth experiments.
Supplementary Fig. 7 One-directional ice growth experiments using Cy5-conjugated PGs
Supplementary Fig. 8 One-directional ice growth with PVA
Supplementary Fig. 9 IRI results of various PGs with varying DB values
Supplementary Fig. 10 Adsorption of PGs on the ice surface
Supplementary Fig. 11 Ice growth simulation of PGs on prismatic ice surface at 267 K
Supplementary Fig. 12 Measurement of z-axis movement of PGs
Supplementary Fig. 13 NMR spin-spin relaxation curve of pure water and PG solutions
Supplementary Fig. 14 Molecular conformational images of multiple H-bonds.
Supplementary Fig. 15 Measurement of hydrodynamics radius
Supplementary Fig. 16 Measurement of 1H DOSY NMR spectra
Supplementary Fig. 17 Hydration Structure of *lin*PG and *hb*PG
Supplementary Fig. 18 Table distribution of multiple hydrogen bonding according to DB
Supplementary Fig. 19 Changes in ice thickness, potential energy and the number of H-bonds formed between *hb*PG and ice over time.
Supplementary Fig. 20 Detachment process of *hb*PG as atop ice layer forms, in regard of the change in the state of H-bonding multiplicity
Supplementary Fig. 21 Lateral movement of PGs at high concentration
Supplementary Fig. 22 Observation of Translocated H_2O molecules using a two-crystal system
Supplementary Fig. 23 Observation of Translocated H_2O molecules using a two-crystal system
Supplementary Fig. 24 Thermal hysteresis of concentrated *hb*PG, *lin*PG and PVA

Supplementary Table 1 Characterization of *hb*PGs with varying degree of branching

Supplementary Table 2 Measurement of T_2 decay and correlation time of *hb*PG and *lin*PG using NMR spin-spin relaxation method.

Supplementary Fig. 1 Synthesis of polyglycerol with controlling DB

a Synthetic scheme of hyperbranched PGs with varying DBs through the copolymerization of glycidol and EEGE with subsequent hydrolysis. L_{13} (linear unit with primary alcohol), L_{14} (linear unit with secondary alcohol), D (dendritic unit), and T (terminal unit). **b**, Deprotection of P(G_{35}-*co*-$EEGE_{59}$) (top, $CDCl_3$, 400 MHz) to *hb*PG (DB = 0.49) (bottom, $CDCl_3$, 400 MHz).

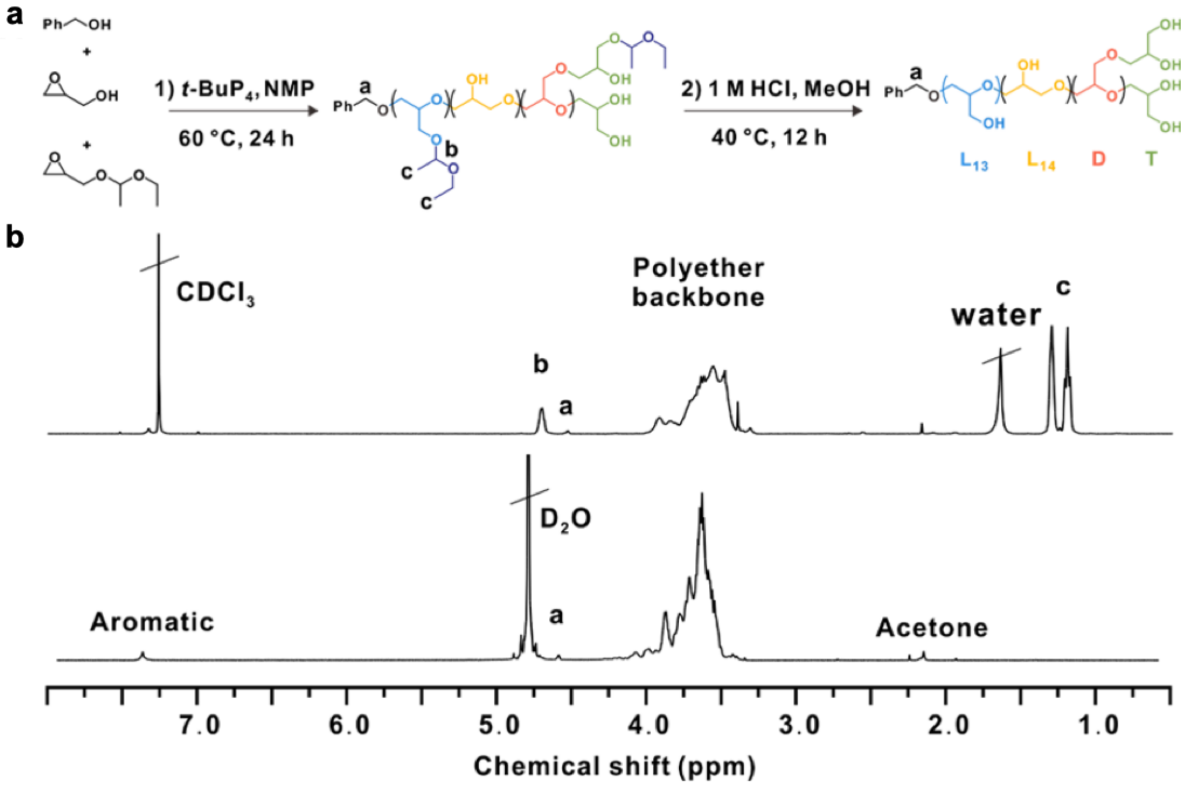

Supplementary Fig. 2 GPC traces of *lin*PG and *hb*PGs

All measurements were performed in DMF using PEG standards (1 mL min^{-1}, 40 °C).

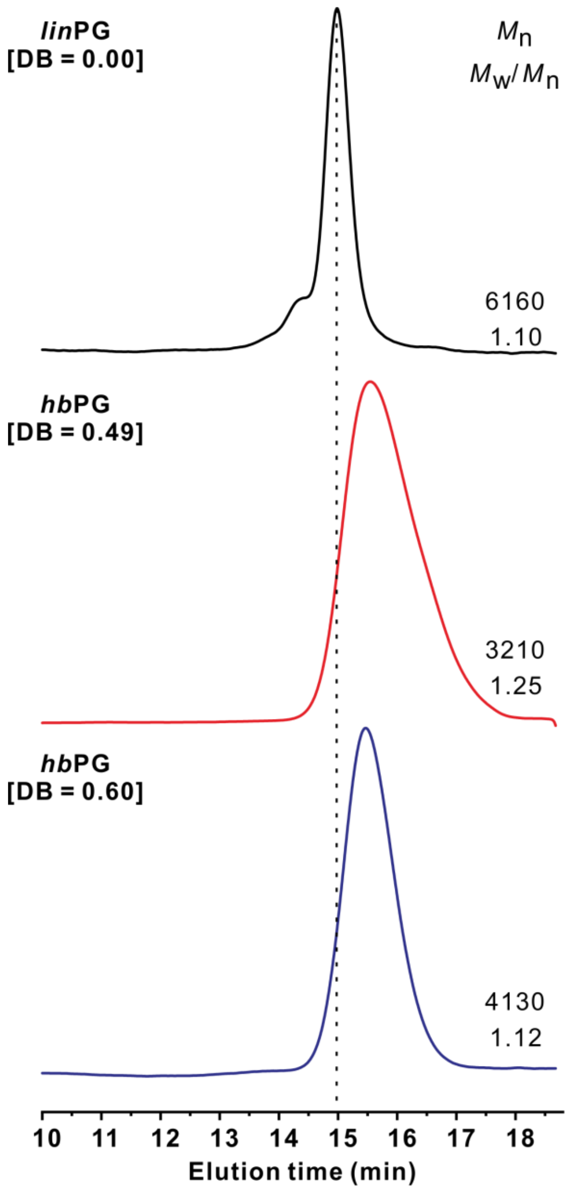

Supplementary Fig. 3 Inverse-gated ^{13}C NMR of PGs

*hb*PG [DB = 0.60], *hb*PG [DB = 0.49], and *lin*PG [DB = 0.60] (101 MHz, D_2O). The blue region (79.6 ppm, 60.8 ppm) represents L_{13} methine carbon, red region (78.2 ppm) represents dendritic (D) methine carbon, yellow region (72.2 ppm) represents L_{14} methine carbon, and the green region (62.7 ppm) represents the carbons on the terminal hydroxyl group.

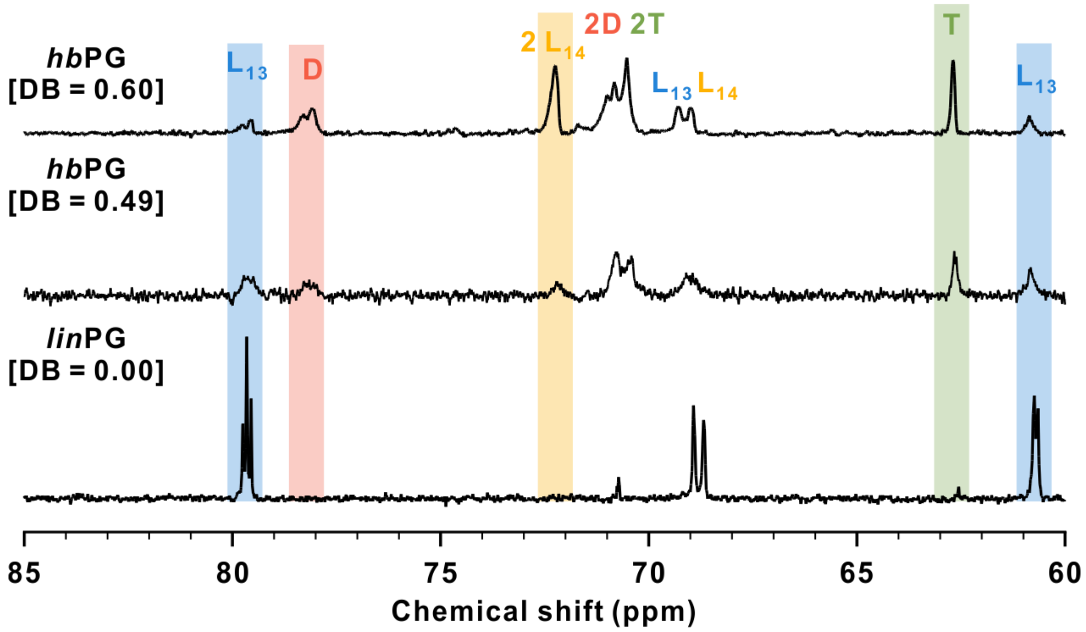

Supplementary Fig. 4 Ratio of each methine groups on PGs

The ratio of polymeric segments was calculated using inverse-gated ^{13}C NMR (101 MHz, D_2O)

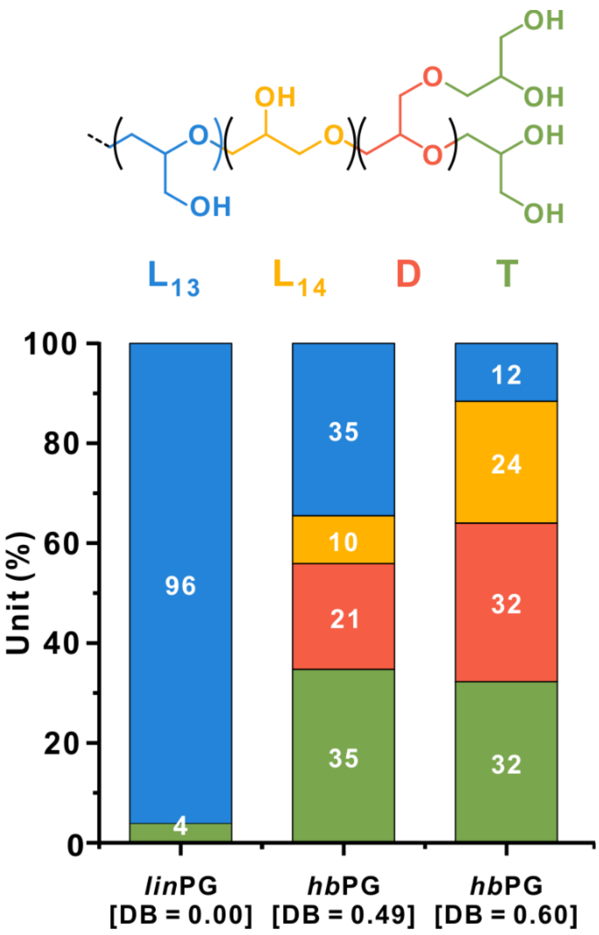

Supplementary Fig. 5 Characterization of Cy5-conjugated PG

a Synthetic scheme of Cy5-conjugated PGs and the representative ^{1}H NMR spectra of Cy5-conjunaged *hb*PG. **b** UV absorbance and photoluminescence (PL) emission spectrum of Cy5-conjugated PG (excitation wavelength: 330 nm). **c**, FT-IR spectrum of Cy5-conjugated *hb*PG.

a

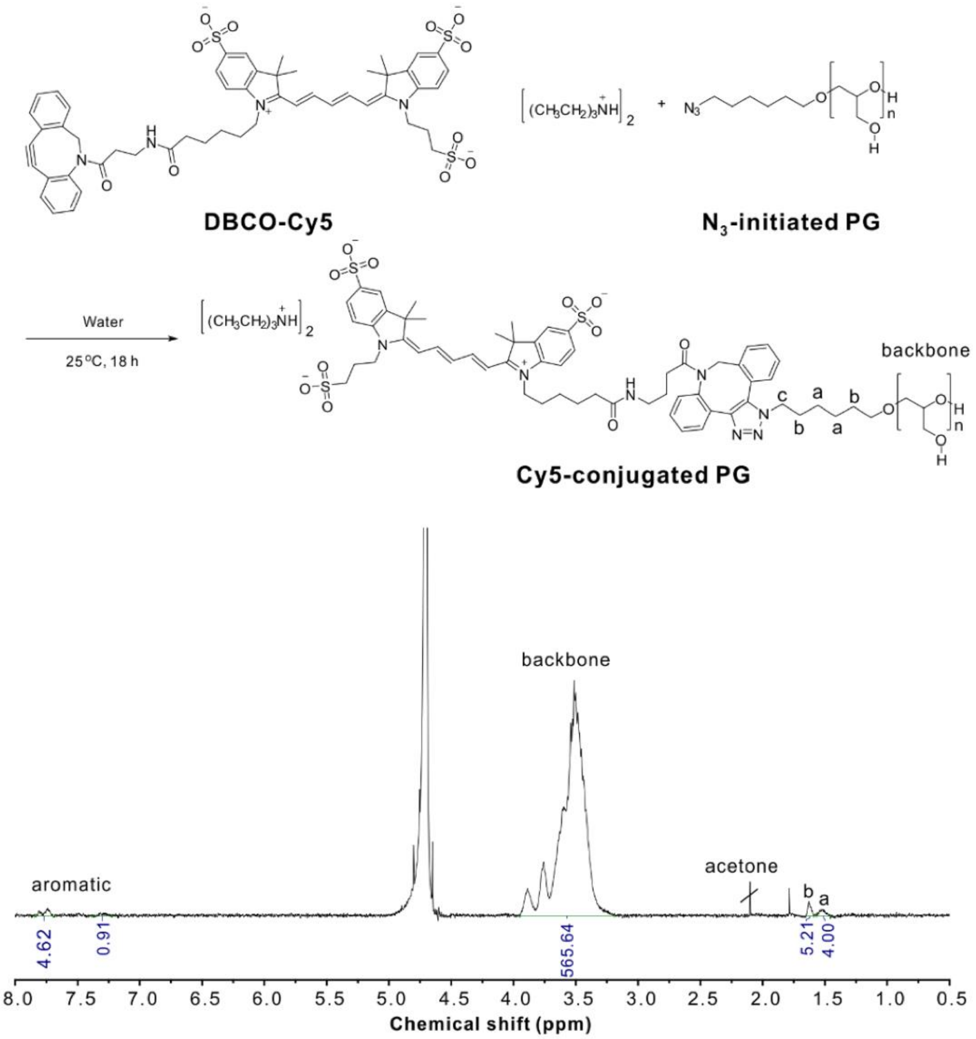

b

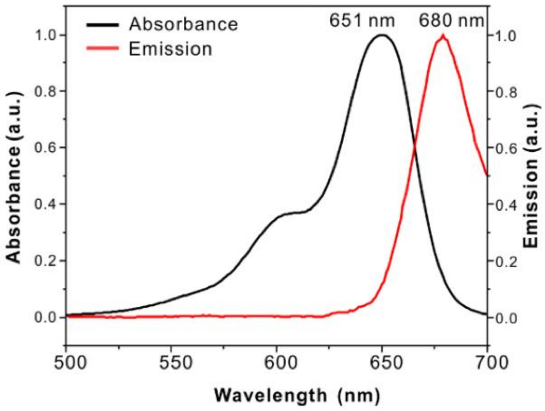

c

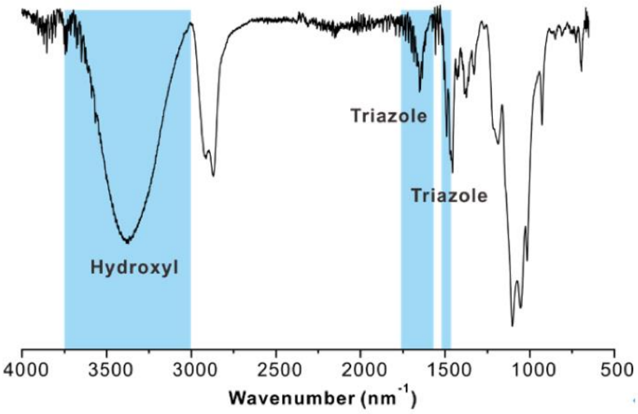

Supplementary Fig. 6 Homemade instrument and environmental conditions for unidirectional ice growth experiments.

a, **b**, Experimental setup of one-directional freezing (**a**) and temperature setting (**b**), which was housed in a cold room maintained at 10 °C. The cold and hot stages consisting of two Peltiers were controlled at 5 °C and -5 °C to maintain a temperature gradient of 3.33°C/mm.

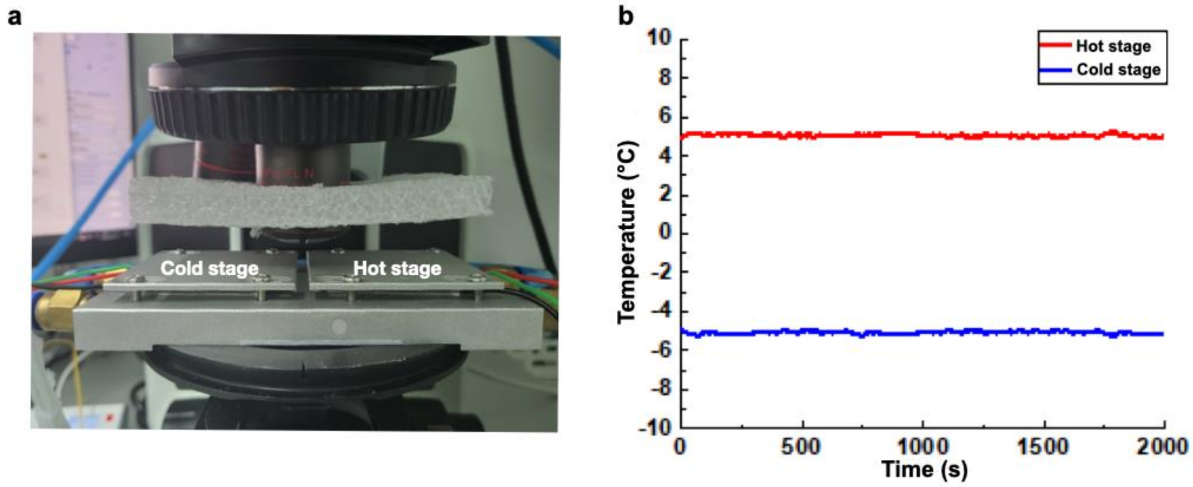

Supplementary Fig. 7 One-directional ice growth experiments using Cy5-conjugated PGs

a, **b**, Fluorescence images when the initial state (first row) and the interface reach the center of the observation window (second row). The images in (**a**) are the result of Cy5-conjugated *hb*PG, while those in (**b**) are the result of Cy5-conjugated *lin*PG.

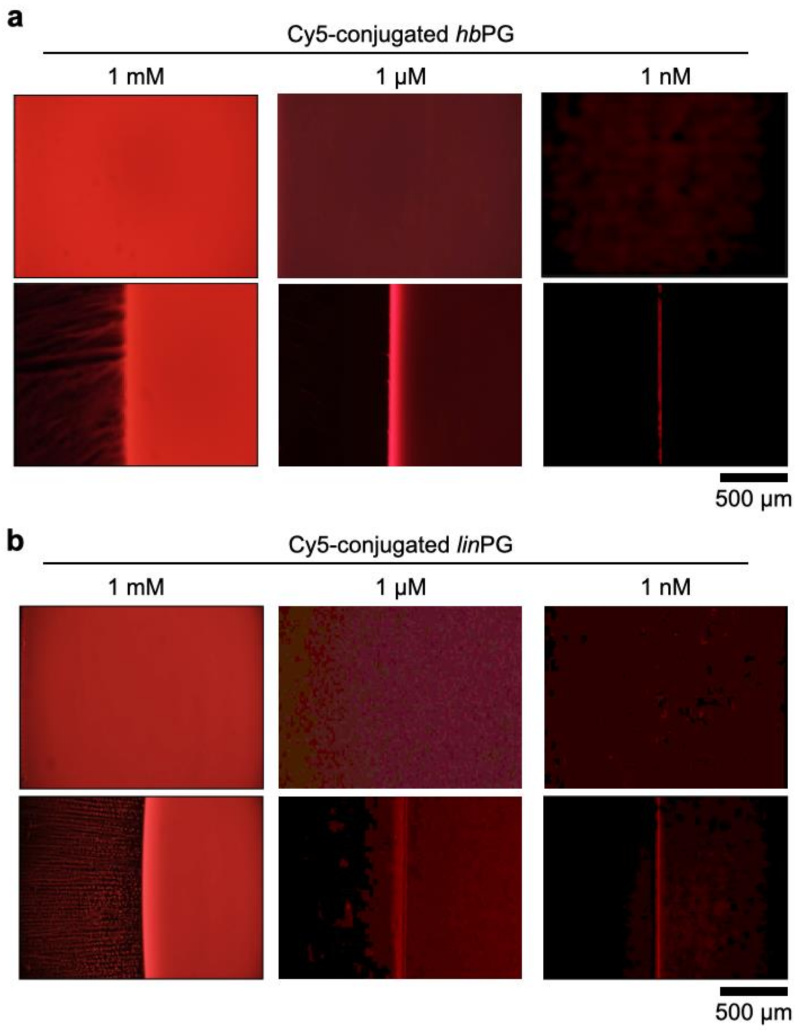

Supplementary Fig. 8 One-directional ice growth with PVA

The ice/water interface moves from left to right over time. Cross-polarized optical images over time for concentrations of 1 mM (left), 1 μM (center), and 1 nM (right). The yellow dashed line indicates the center position of the observation range. PVA was purchased from Sigma-Aldrich (M_w = 9,000–10,000 Da).

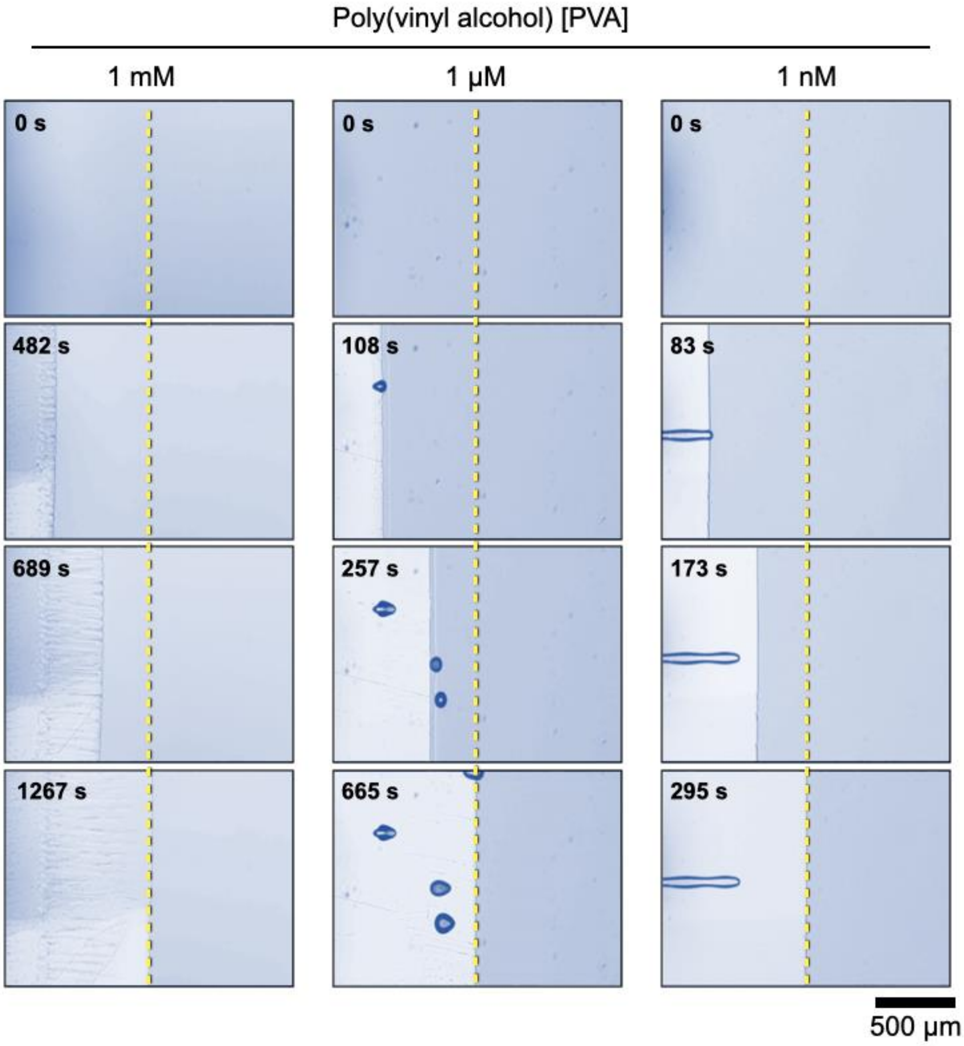

Supplementary Fig. 9 IRI results of various PGs with varying DB values

The RI values of *lin*PG [DB = 0.00], *hb*PG [DB = 0.49], and *hb*PG [DB = 0.60] are indicated by green, orange, and red hollow circles, respectively. For each substance, the concentration was decreased by 10 times from 1 mM to 10^{-5} mM (10 nM), and the average value was calculated from four independent measurements with standard deviation. Each data point is indicated by a solid eye-guiding line.

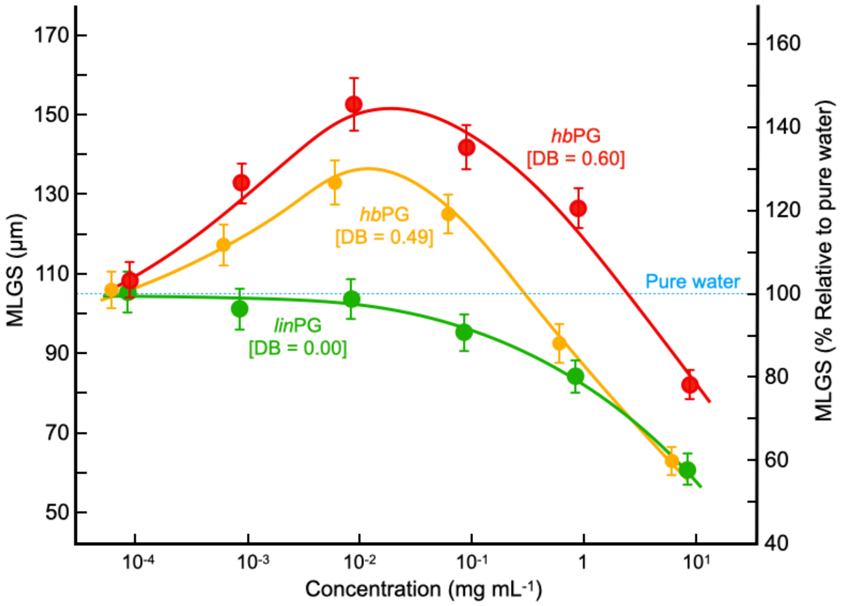

Supplementary Fig. 10 Adsorption of PGs on the ice surface

Change of binding Gibbs free energy (potential of mean force, PMF) calculated from umbrella sampling simulations along the distance between ice surface and the PGs (*hb*PG-red line, *lin*PG-green line).

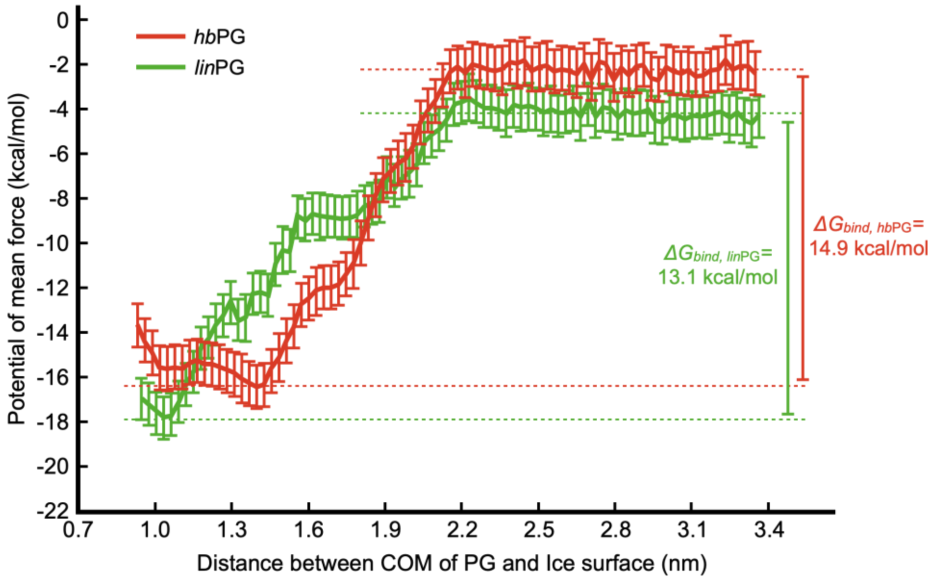

Supplementary Fig. 11 Ice growth simulation of PGs on prismatic ice surface at 267 K

a, Final configuration (i.e., 300 ns) of ice growth simulation of pure water (first column), *hb*PG at low (second column) and high (third column) concentrations, and *lin*PG at low (fourth column), and high (fifth column) concentrations. **b**, Changes in ice thickness and number of H-bonds formed with ice on prismatic plane. **c,** Same as (**b**), but at higher concentrations of *hb*PG and *lin*PG.

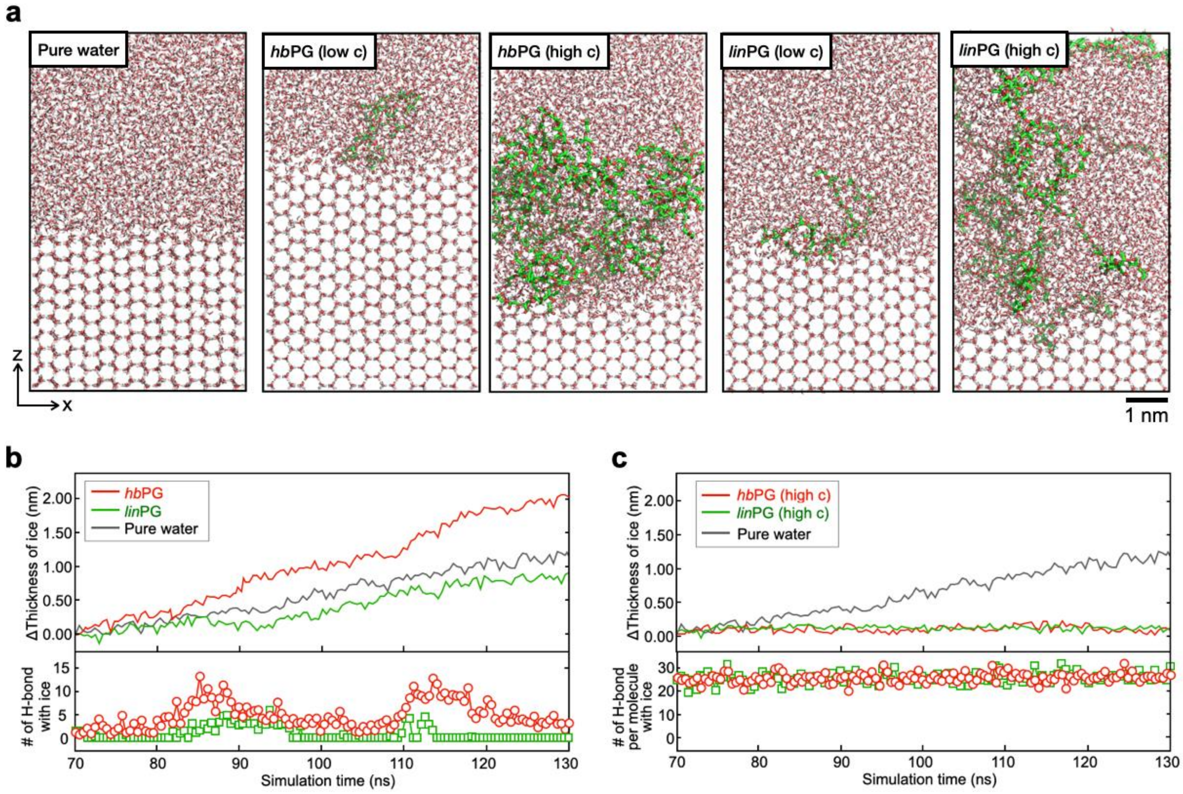

Supplementary Fig. 12 Measurement of z-axis movement of PGs

The antifreeze activity was evaluated by calculating the height of *lin*PG [DB = 0.00] (green line), *hb*PG [DB = 0.49] (orange line), and *hb*PG [DB = 0.60] (red line).

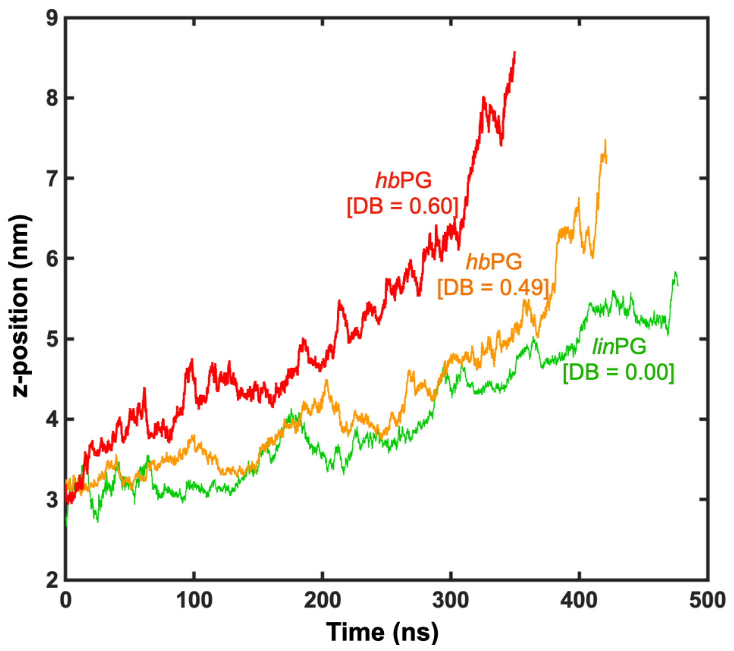

Supplementary Fig. 13 NMR spin-spin relaxation curve of pure water and PG solutions

T_2 decay curves of water molecules fitted by one exponential function for pure water (black symbols). *hb*PG (red symbols) and *lin*PG (green symbols) fitted by a biexponential function at 25°C.

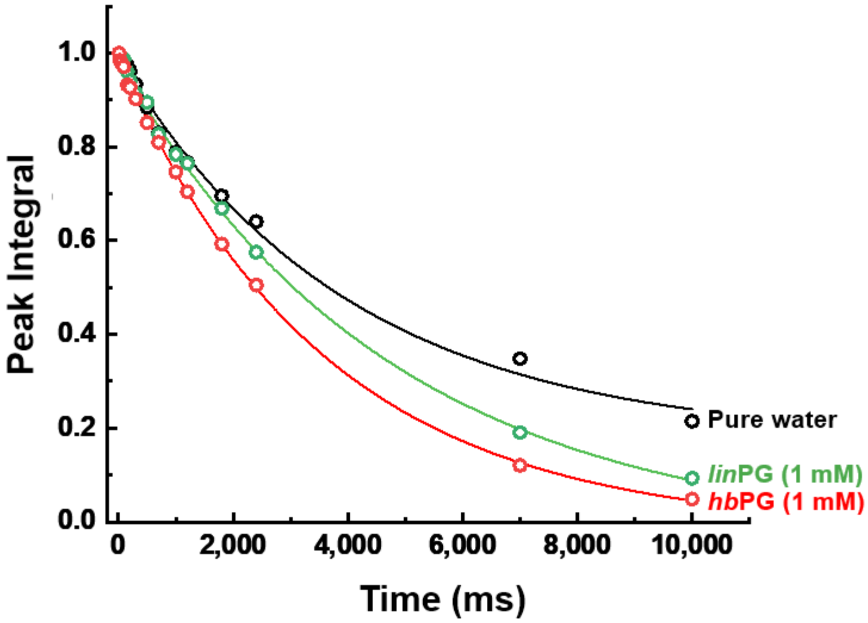

We measured NMR spin-spin relaxation (T_2) for PGs. The T_2 relaxation behaviors for the water proton were fitted with a biexponential decay curve (equation 1)

$$E_t = f_{2,a} exp\left(-\frac{t}{T_{2,a}}\right) + f_{2,b} exp\left(-\frac{t}{T_{2,b}}\right) + E_0 \cdots\cdots\cdots\cdots (equation\ 1)$$

The dynamic of water can be represented by the correlation time for the motion of water (τ_c) by using the Bloembergen Purcell and Pound equation (equation 2).

$$\frac{1}{T_2} = \frac{C}{2}(3\tau_C + \frac{5\tau_C}{1+\omega_0{}^2\tau_C{}^2} + \frac{5\tau_C}{1+4\omega_0{}^2\tau_C{}^2}) \cdots\cdots\cdots\cdots (equation\ 2)$$

Supplementary Fig. 14 Molecular conformational images of multiple H-bonds.

Multiple hydrogen bonds are formed between a water molecule and PG molecule. Oxygen, hydrogen, and carbon atoms are represented by red, white, and green sticks, respectively. The yellow dashed line indicates hydrogen bonding.

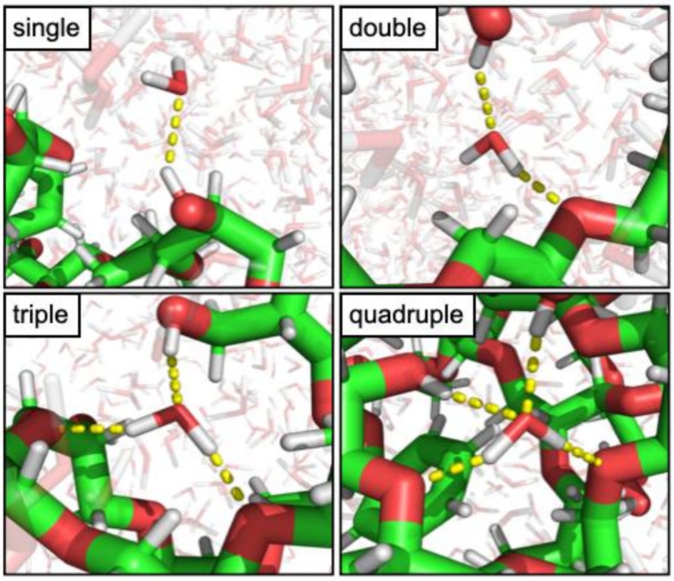

Supplementary Fig. 15 Measurement of hydrodynamics radius

Hydrodynamic radius (R_h) of (a) *hb*PG and (b) *lin*PG at 10 µM, 100 µM and 1 mM concentration measured by DLS.

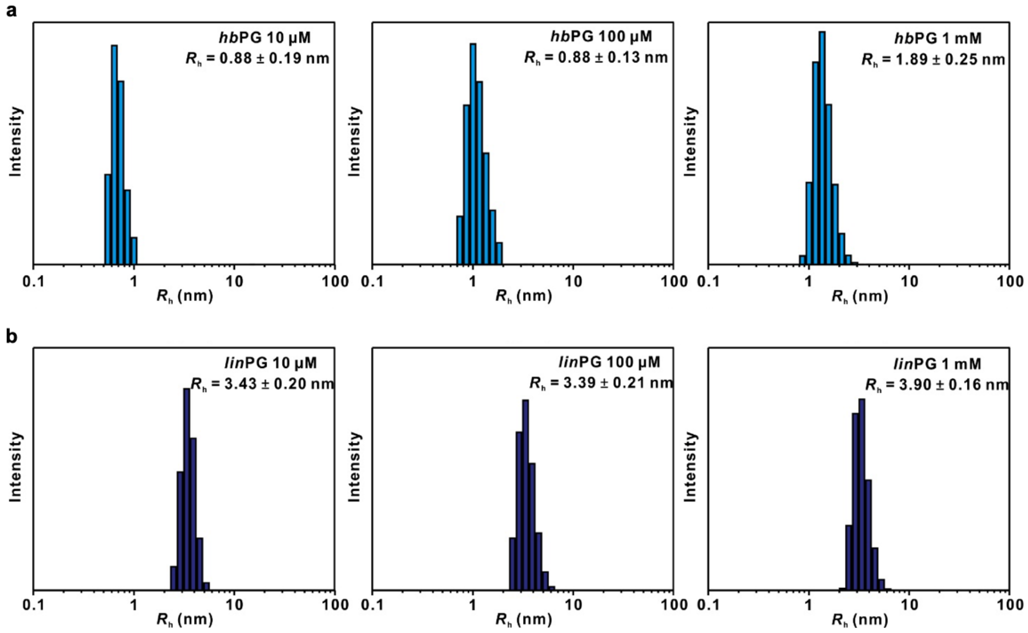

Supplementary Fig. 16 Measurement of 1H DOSY NMR spectra
(a) Stejskal–Tanner plot obtained from ^{1}H DOSY spectra with the corresponding diffusion coefficient (*D*) values. (b) ^{1}H DOSY NMR spectrum of *hb*PG$_{100}$ (D_2O, 25 °C). (c) ^{1}H DOSY NMR spectrum of *lin*PG$_{100}$ (D_2O, 25 °C).

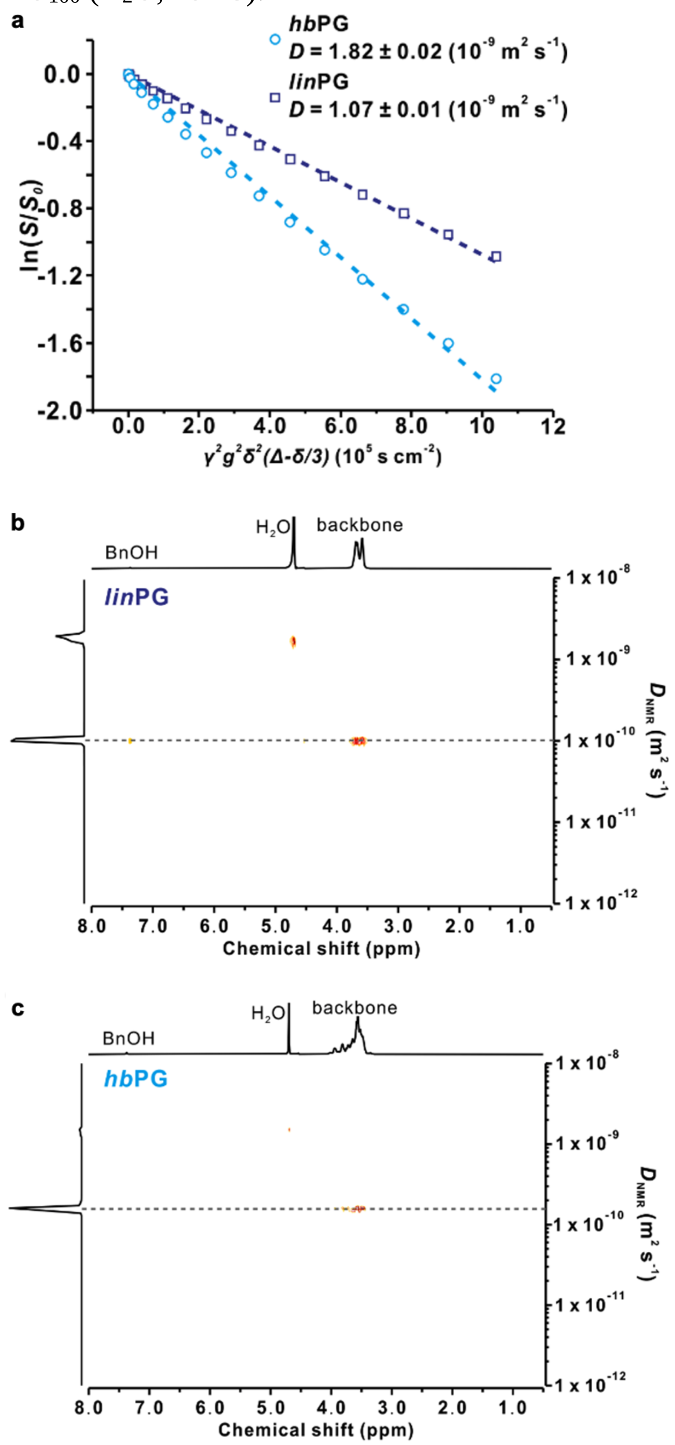

Supplementary Fig. 17 Hydration Structure of *lin*PG and *hb*PG

a, Gibbs free energy profile as a radius of gyration of *lin*PG. **b,** Time-traced conformational change in the R_g of *lin*PG as initial structures of four different lengths. **c,** same as (**b**), but for *hb*PG with R_g of 0.9 nm.

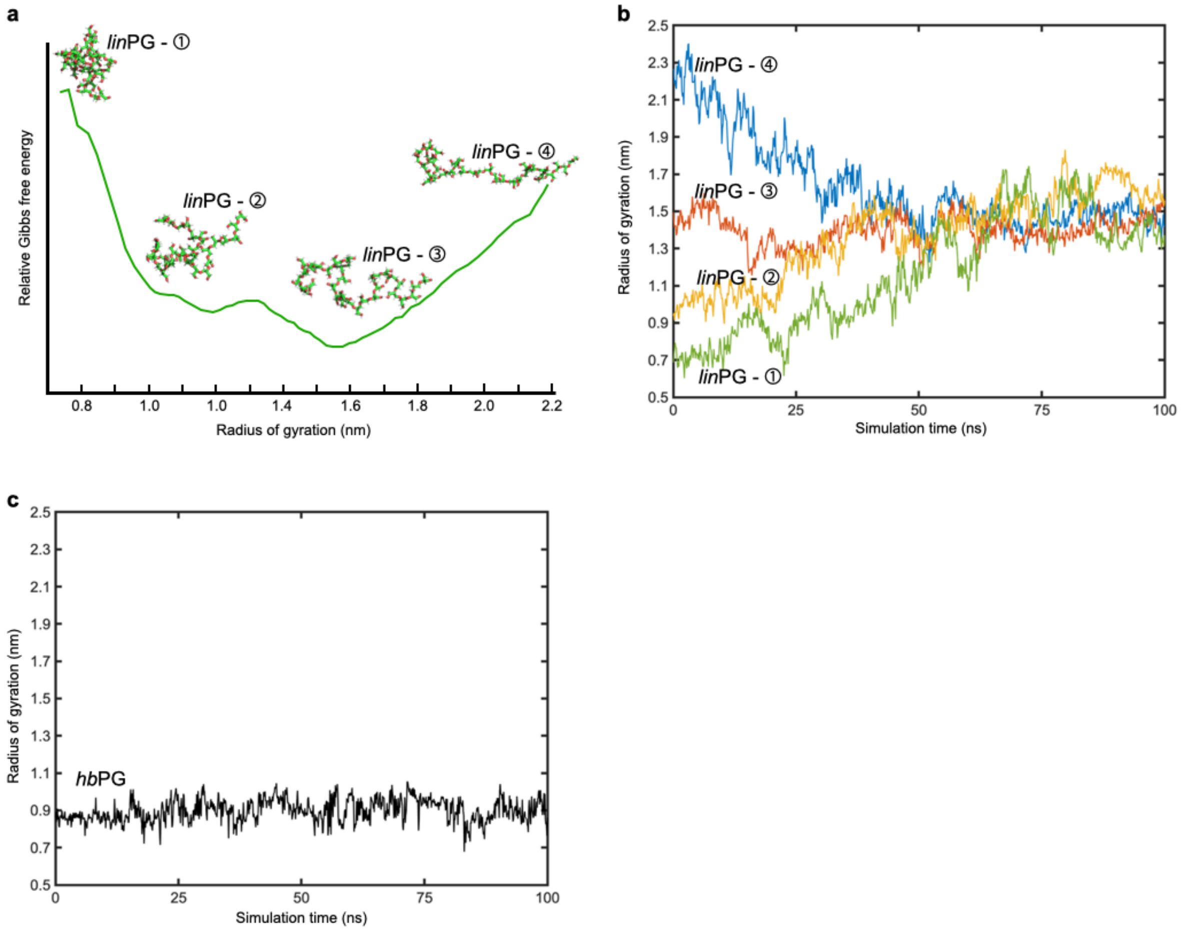

Supplementary Fig. 18 Table distribution of multiple hydrogen bonding according to DB

a, **b**, **c**, From the back-tracking analysis, the hydrogen bonds between the H_2O molecule and PG's segments were classified into (**a**) double, (**b**) triple, and (**c**) quadruple bonding types. The double bond is a form in which a H_2O molecule bonds with two segments of PG. The triple bond was designated as a bond formed between a H_2O molecule and two units and an additional D group (denoted by +D), while the quadruple bond included another D group (denoted by +2D).

a

*hb*PG [DB = 0.60]

	L_{13}	L_{14}	D	T
L_{13}	2			
L_{14}	8	3		
D	11	14	24	
T	5	7	26	8

*hb*PG [DB = 0.49]

	L_{13}	L_{14}	D	T
L_{13}	5			
L_{14}	2	1		
D	2	5	10	
T	2	8	9	5

*lin*PG [DB = 0.00]

	L_{13}	L_{14}	D	T
L_{13}	10			
L_{14}	0	0		
D	0	0	0	
T	1	0	0	0

b

*hb*PG [DB = 0.60]

+D	L_{13}	L_{14}	D	T
L_{13}	0			
L_{14}	1	0		
D	5	6	0	
T	1	2	2	1

*hb*PG [DB = 0.49]

+D	L_{13}	L_{14}	D	T
L_{13}	0			
L_{14}	1	0		
D	3	2	0	
T	0	1	1	0

*lin*PG [DB = 0.00]

+D	L_{13}	L_{14}	D	T
L_{13}	1			
L_{14}	0	0		
D	0	0	0	
T	0	0	0	0

c

*hb*PG [DB = 0.60]

+2D	L_{13}	L_{14}	D	T
L_{13}	0			
L_{14}	1	1		
D	0	0	0	
T	0	0	0	0

*hb*PG [DB = 0.49]

+2D	L_{13}	L_{14}	D	T
L_{13}	0			
L_{14}	0	0		
D	0	0	0	
T	0	0	0	0

*lin*PG [DB = 0.00]

+2D	L_{13}	L_{14}	D	T
L_{13}	0			
L_{14}	0	0		
D	0	0	0	
T	0	0	0	0

Supplementary Fig. 19 Changes in ice thickness, potential energy and the number of H-bonds formed between *hb*PG and ice over time.

a, (**a,** top) Time-traced changes in ice thickness and potential energy from 70 to 130 ns. The changes in ice thickness and potential energy are represented by red and gray lines, respectively. (**a**, bottom) The number of hydrogen bonds between *hb*PG and ice, denoted by red circles, was calculated during the same time period. Four moments, that is, (1) to (4), were marked to investigate the dynamics of the growth of ice layer #2. **b**, The molecular configuration corresponding to each moment is shown in (**b**): At the moment (1) *hb*PG is positioned above the ice surface after layer #1 is formed, (2) structured H_2O molecules are induced by *hb*PG, (3) epitaxial ice growth occurs to form ice layer #2, and (4) *hb*PG dissociated hydrogen bonds with ice.

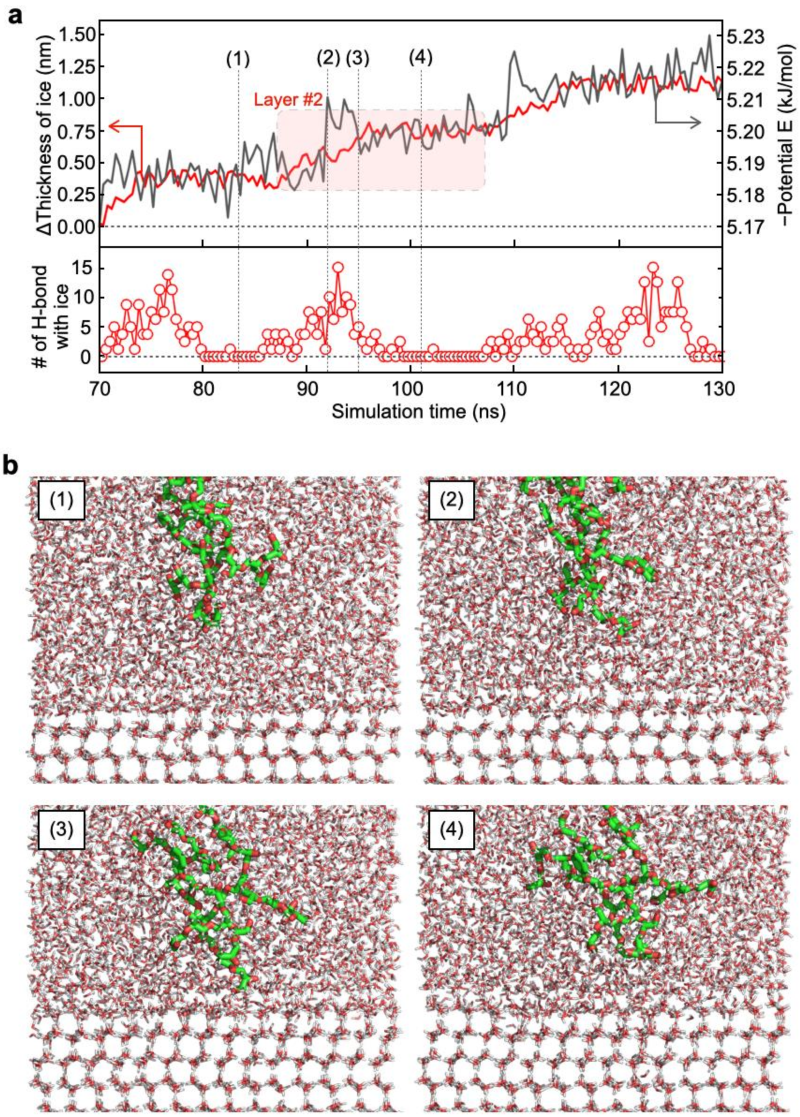

Supplementary Fig. 20 Detachment process of *hb*PG as atop ice layer forms, in regard of the change in the state of H-bonding multiplicity

Trajectory tracing of water molecules that had formed multiple hydrogen bonding with *hb*PG. Hydrogen bonds formed with water molecules selected in magenta are indicated by yellow dotted lines.

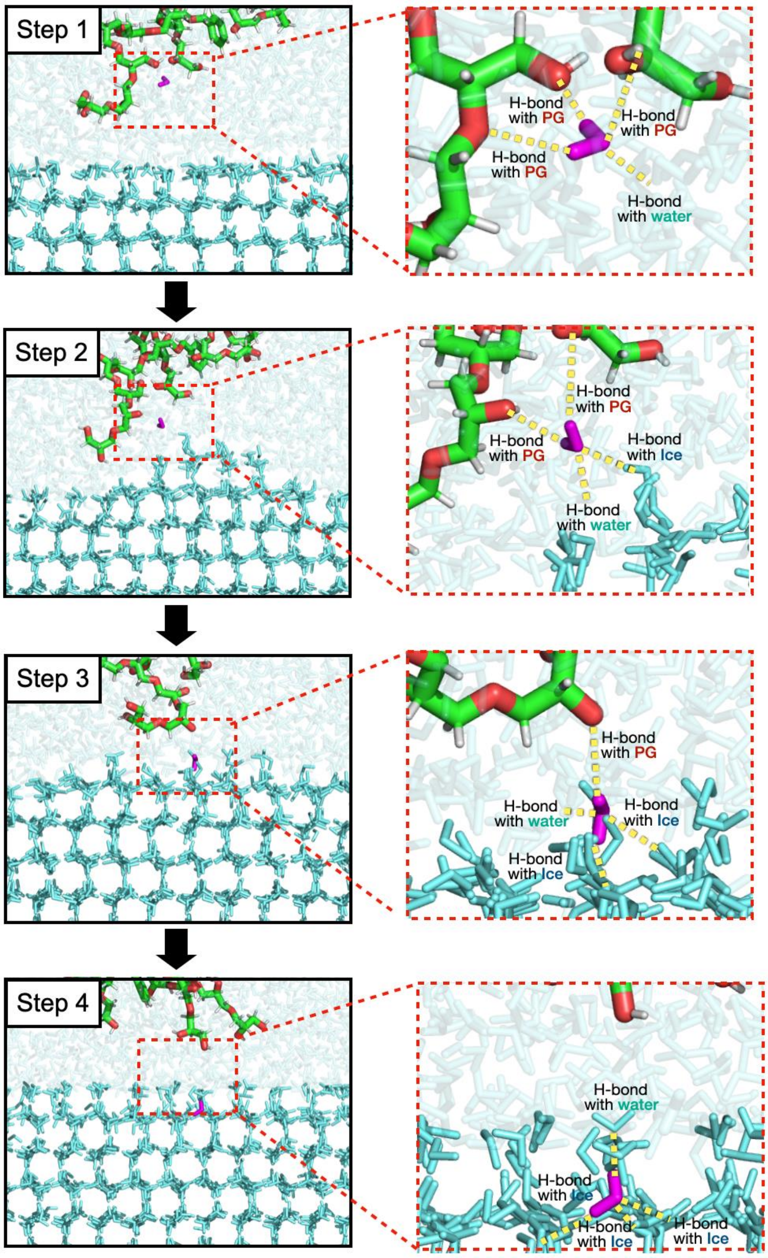

Supplementary Fig. 21 Lateral movement of PGs at high concentration

The lateral movements of concentrated *hb*PG (red line) and *lin*PG (green line) for 60 ns, starting from the position at 70 ns to that at 130 ns, were calculated. Each movement was observed for the 10 PG molecules present in the simulation system.

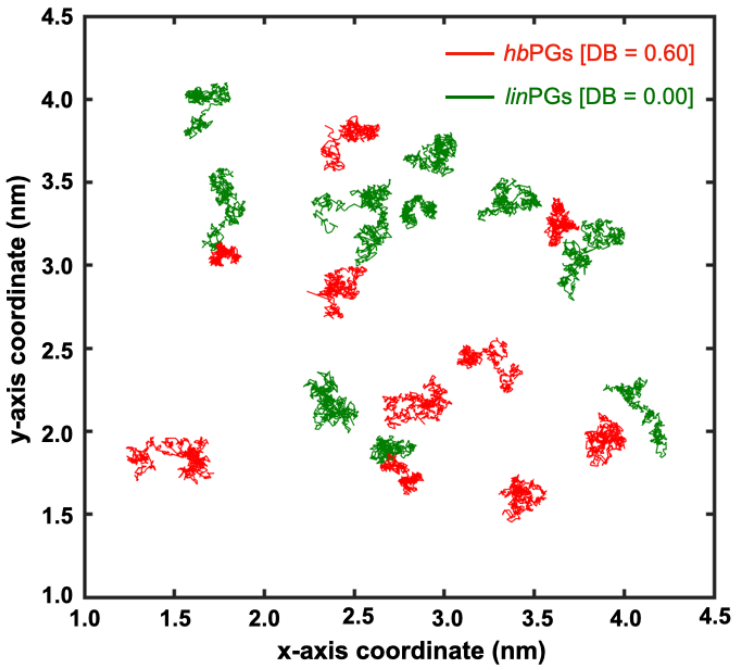

Supplementary Fig. 22 Observation of Translocated H_2O molecules using a two-crystal system

Snapshot of MD simulation for (a) low concentration and (b) high concentration of *hb*PG. The blue spheres represent H_2O molecules from the top crystal and orange spheres represents from the bottom crystal. (c) The number of H_2O molecules translocated to another crystal in high concentrated *hb*PG (red symbol) and low concentrated *hb*PG (black symbol) per 1 ns interval.

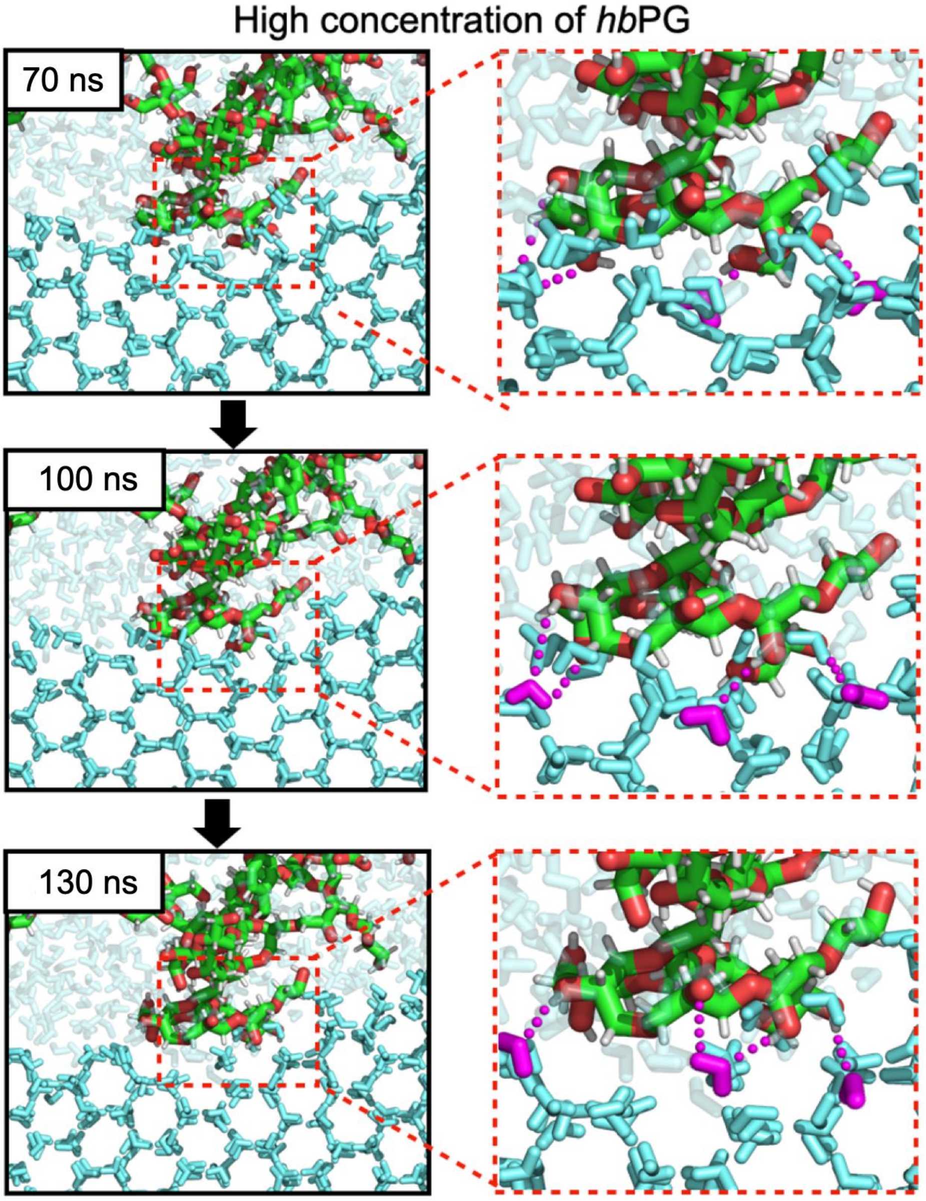

Supplementary Fig. 23 Observation of Translocated H_2O molecules using a two-crystal system

Snapshot of MD simulation for (a) low concentration and (b) high concentration of *hb*PG. The blue spheres represent H_2O molecules from the top crystal and orange spheres represents from the bottom crystal. (c) The number of H_2O molecules translocated to another crystal in high concentrated *hb*PG (red symbol) and low concentrated *hb*PG (black symbol) per 1 ns interval.

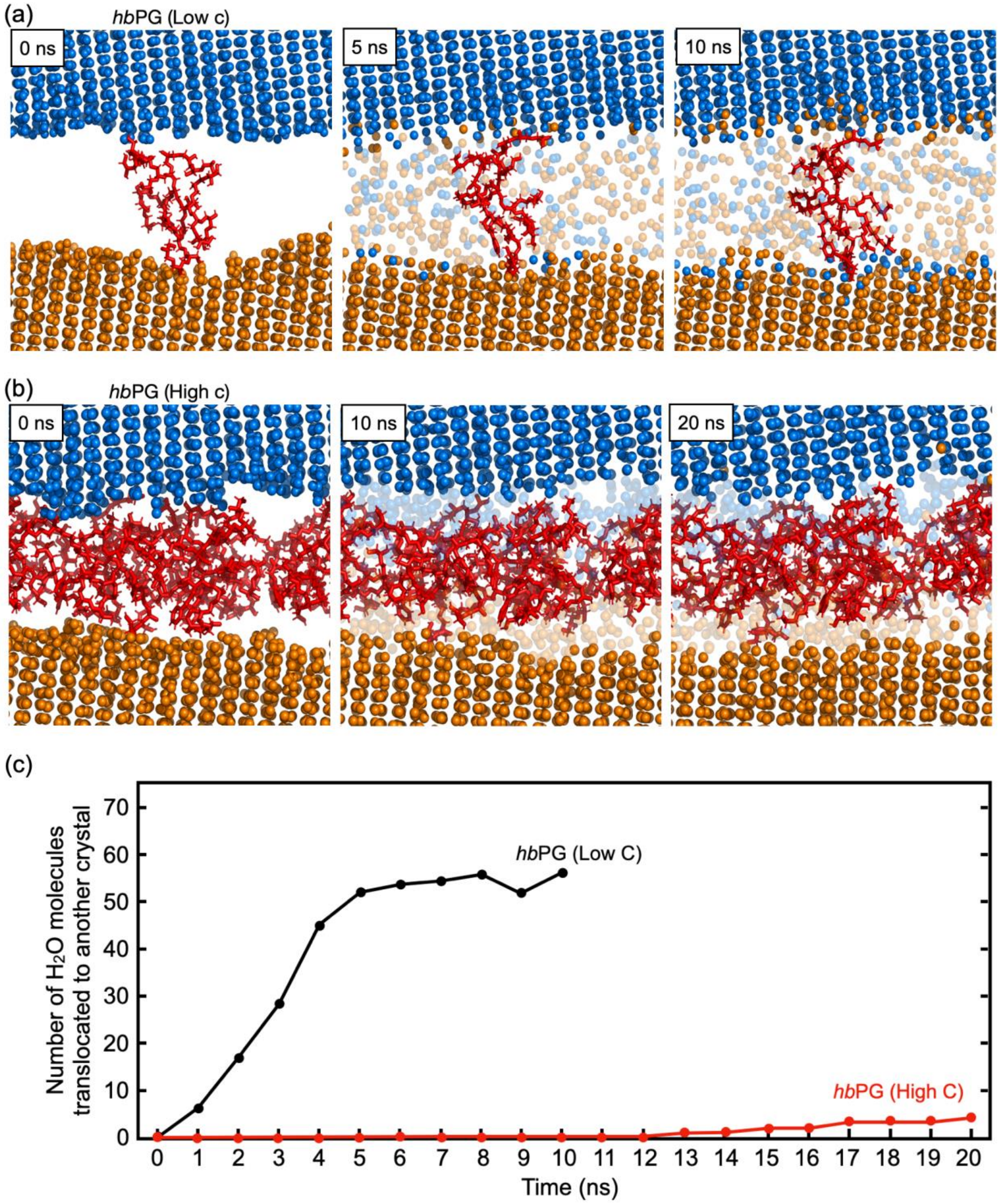

Supplementary Fig. 24 Thermal hysteresis of concentrated *hb*PG, *lin*PG and PVA
Optical microscopic images of ice crystals with diameters of 10, 20, 35 and 50 µm.

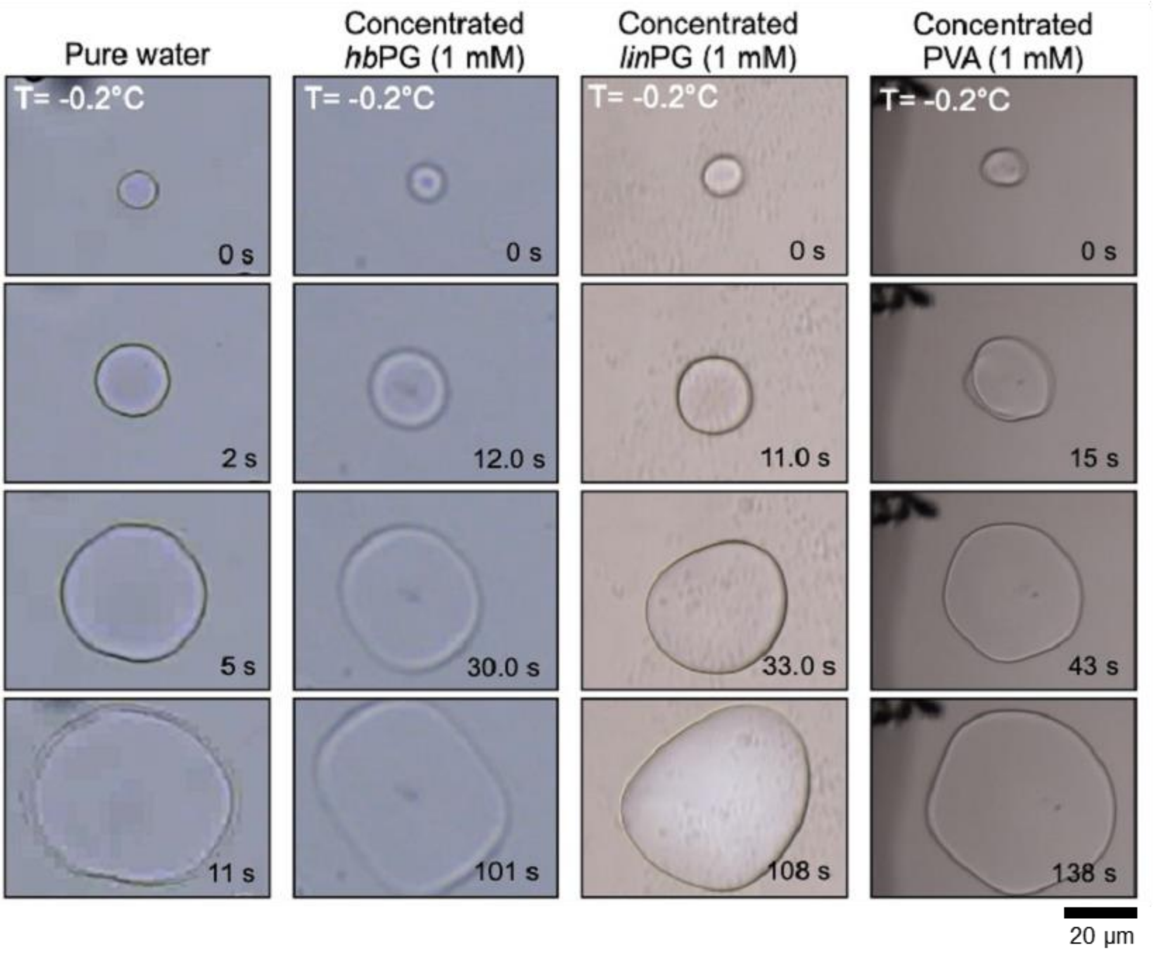

Supplementary Table 1 Characterization of *hb*PGs with varying degree of branching

[a] calculated from inverse-gated ^{13}C NMR. [b]$M_{n,NMR}$, and composition of the copolymer were determined via ^{1}H NMR in D_2O, [c]$M_{n,GPC}$, and Đ was measured by GPC analysis with PEG standards in DMF.

	Entry	DB[a]	Composition	$M_{n,NMR}$[b] (g mol^{-1})	$M_{n,GPC}$[c] (g mol^{-1})	*Đ*[c]
linPG	1	0.00	*lin*PG$_{114}$	8480	6160	1.10
hbPG	2	0.49	*hb*PG$_{81}$	6120	3210	1.25
	3	0.60	*hb*PG$_{118}$	8900	4130	1.12

Supplementary Table 2 Measurement of T_2 decay and correlation time of *hb*PG and *lin*PG using NMR spin-spin relaxation method.

	*hb*PG (1 mM)	*lin*PG (1 mM)
$T_{2,BW}$	4046 ms	3622 ms
$\tau_{c,BW}$	1.43×10^{-11} ms	1.59×10^{-11} ms
$T_{2,CW}$	84 ms	1569 ms
$\tau_{c,CW}$	60.79×10^{-11} ms	3.68×10^{-11} ms
