# Supplementary material for: Janus regulation of ice growth by hyperbranched polyglycerols generating dynamic hydrogen bonding
Source: Nat Commun. 2022 Nov 1;13:6532. doi: 10.1038/s41467-022-34300-x (PMC9626502; doi:10.1038/s41467-022-34300-x)
Supplement: Supplementary file 2 — Description of Additional Supplementary Files [file 41467_2022_34300_MOESM2_ESM.pdf]

Description of Additional Supplementary Files

File Name: Supplementary Movie 1
Description: Dynamic motion of low- and high-concentrated hbPGs and H_2O molecules during ice growth at 267 K for 300 ns

File Name: Supplementary Movie 2
Description: Dynamic motion of low- and high-concentrated linPGs and H_2O molecules during ice growth at 267 K for 300 ns

File Name: Supplementary Movie 3
Description: Reverse-regeneration of ice growth with hbPG for back-tracking analysis

File Name: Supplementary Movie 4
Description: Reverse-regeneration of ice growth with linPG for back-tracking analy
